# Nanodiamond-Based Spatial–Temporal Deformation Sensing for Cell Mechanics

**DOI:** 10.1021/acsnano.4c15003

**Published:** 2025-04-02

**Authors:** Yue Cui, Weng-Hang Leong, Guoli Zhu, Ren-Bao Liu, Quan Li

**Affiliations:** † Department of Physics, The Chinese University of Hong Kong, Shatin, New Territories, Hong Kong 999077, China; ‡ Quantum Science Center of Guangdong-Hong Kong-Macao Greater Bay Area (Guangdong), Shenzhen 518045, China; § Department of Engineering Science, Faculty of Innovation Engineering, Macau University of Science and Technology, Taipa, Macao 999078, China; ∥ Centre for Quantum Coherence, The Chinese University of Hong Kong, Shatin, New Territories, Hong Kong 999077, China; ⊥ State Key Laboratory of Quantum Information Technologies and Materials, The Chinese University of Hong Kong, Shatin, New Territories, Hong Kong 999077, China; # New Cornerstone Science Laboratory, The Chinese University of Hong Kong, Shatin, New Territories, Hong Kong 999077, China

**Keywords:** spatial–temporal mechanical analysis, dynamic nonlocal deformation, nitrogen-vacancy centers in nanodiamond, optically detected magnetic resonance, viscoelasticity of cells, elastocapillary effect

## Abstract

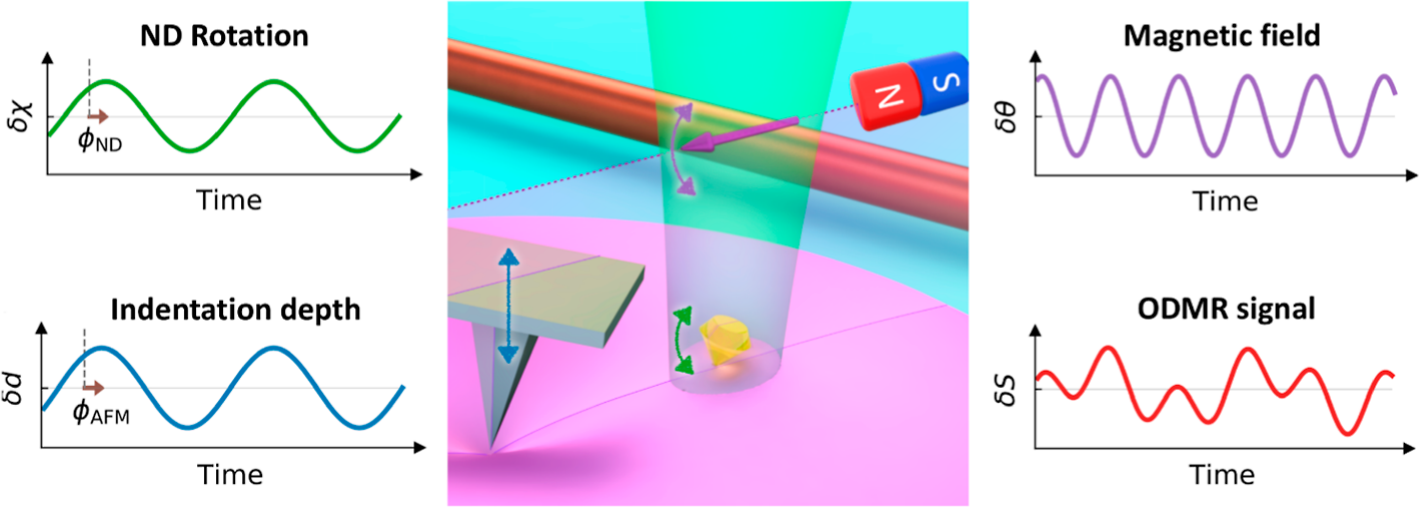

Precise assessment of the mechanical properties of soft biological systems at the nanoscale is crucial for understanding physiology and pathology and developing relevant drugs. Conventional atomic force microscopy (AFM)-based indentation methods suffer from uncertainties in local tip–sample interactions and the model choice. This can be overcome by adopting spatially resolved nonlocal deformation sensing for mechanical analysis. However, the technique is currently limited to lifeless/static systems due to the inadequate spatial or temporal resolution or difficulties in differentiating the indentation-induced deformation from that associated with live activities and other external perturbations. Here, we develop a dynamic nonlocal deformation sensing approach allowing both spatially and temporally resolved mechanical analysis, which achieves a tens of microsecond time-lag precision, a nanometer vertical deformation precision, and a subhundred nanometer lateral spatial resolution. Using oscillatory nanoindentation and spectroscopic analysis, the method can separate the indentation-caused signal from random noise, enabling a live cell measurement. Using this method, we discover a distance-dependent phase of surface deformation during indentation, leading to the disclosure of surface tension effects (capillarity) in the mechanical response of viscoelastic materials and live cells upon AFM indentation. A viscoelastic model with surface tension is used to enable simultaneous quantification of the viscoelasticity and capillarity of the cell. We show that neglecting surface tension, as in conventional AFM methods, would underestimate the liquid-like characteristics and overestimate the apparent viscoelastic modulus of cells. This study provides exciting opportunities to understand a broad range of elastocapillarity-related interfacial mechanics and mechanobiological processes in live cells.

## Introduction

Cell mechanical properties are tightly associated with a variety of cellular processes, including division,^1^ differentiation,^2^ diffusion,^3^ and motility,^4^ and are important emerging biomarkers for cancer detection, diagnosis, and classification.^5−7^ Various methods such as atomic force microscopy (AFM) and magnetic/optical tweezer, which allows dynamic measurement of mechanical forces and deformations at the single cell level, have advanced our understanding of the frequency-dependent cell viscoelasticity and rheological behaviors over a wide frequency range.^5,7,8^ However, these techniques based on force-deformation measurement at the local indentation position still face several limitations in the actual cell mechanical characterization. On one hand, relating the data to quantitative mechanical properties relies on correct modeling and requires detailed knowledge about the local contact.^9,10^ On the other hand, the methods provide no information about the nonlocal (spatial) response of cells to local loading. The nonlocal information could be useful for observing important mechanical information related to cell interface such as the capillary action and surface tension effect.^11,12^

Nonlocal deformation measurement, in which the deformation is measured at a location away from where the deformation is induced (e.g., by nanoindentation), is advantageous for analyzing the intrinsic mechanical properties of materials, since it is unaffected by complications at the local contacts.^13^ The past decade has seen revolutionary advances in development of methods for measuring deformation with nonlocal information, based on optical imaging^14,15^ or deformation reconstruction from sensor rotation.^10,11^ Recent works in this emerging field have shown that the usually overlooked surface tension in the solid can play a central role in the mechanics of soft solids such as polymer gels^12^ and elastomers^10,16^ and can affect the evaluation of the elastic modulus of fixed cells by AFM indentation.^11^ Despite the importance of interfacial mechanics in regulating cellular processes,^17−19^ the influence of surface tension (capillarity) in the mechanical responses of live cells upon AFM indentation remains unclear due to the challenges in applying the nonlocal deformation sensing methods in live cells. Typically limited by optical wavelength, optical imaging of the surface deformation profile does not have sufficient precision and resolution. For deformation measurements on live systems, nanometer precision is needed for less-invasive (hence shallow) indentation. Additionally, nanoscale lateral spatial resolution is required since the critical elastocapillary lengths related to the elastocapillary phenomena of biological systems, defined as the ratio of surface tension to bulk Young’s modulus, are usually in the order of micron.^11^ The nonlocal deformation sensing methods based on nanodiamond (ND) rotation sensing features nanometer precision as well as subhundred nanometer spatial resolution,^10^ but the technique is currently restricted to lifeless and static systems, since it cannot differentiate indentation-induced material deformation from that originating from live-associated activities.^11^ It also lacks information about the temporal mechanical response of materials and is incapable of capturing material viscoelasticity.

In this work, we develop a diamond-based dynamic nonlocal deformation sensing scheme with high precision and high spatial and temporal resolution, which enables both spatially and temporally resolved mechanical analysis of soft materials and live cells. We overcome the limitation of the diamond-based deformation sensing to static systems by combining oscillatory AFM nanoindentation^5,8^ with frequency-domain measurement of the induced deformation. The dynamic deformation is converted to periodically variations in the orientation of the nitrogen-vacancy (NV) centers in diamond through the diamond sensors preanchored on the material surface. Based on the established quantum sensing protocols for orientation measurement of NV centers, we develop a fast rotation sensing method using a two-point optically detected magnetic resonance (ODMR) measurement with a high duty ratio. We realize phase-sensitive detection of the ND rotation by synchronizing the AFM indentation and fast rotation measurement. The signals at the oscillation frequency single out the indentation-induced deformation from that induced by other sources such as rotation diffusion and cell activities.^11,20,21^ An additional benefit of the alternating current (AC) measurement is that it filters out the background fluorescence noise and therefore enhances the signal-to-noise ratio and enables the deformation measurement in complex live systems.^22^

We demonstrate the scheme by measuring viscoelastic polydimethylsiloxane (PDMS) films and live MCF-7 cells. Through the phase-sensitive spectral analysis, we achieve 0.01π precision in phase lag measurement (tens of microsecond time-lag precision), ∼2 nm vertical deformation precision, and subhundred nanometer lateral spatial resolution in the dynamic nonlocal deformation mapping. Our experiments led to the first discovery of a distance-dependent phase lag of the nonlocal deformation during the oscillatory indentation. The phase lag reveals the elastocapillary effects in the mechanical response, indicating that the bulk viscoelasticity (dominant at larger scales) and the surface tension (dominant at smaller scales) are competing factors when the lateral length scales of the deformation are comparable to the elastocapillary length of the material (typically in the order of micrometers for soft materials). The interplay between the bulk and surface responses in the nonlocal deformation enables simultaneous assessment of the surface tension (capillarity) and the viscoelasticity [quantified by a frequency-dependent complex modulus , where the real part  and the imaginary part  are the storage and loss moduli, respectively, and the loss angle δ_loss_ varies between 0 and π/2 with 0 representing a complete solid and π/2 a complete liquid]. We show that an overlook of the surface tension, as in conventional local AFM-indentation measurement using classical contact models, would lead to an overestimation of the magnitude () and an underestimation of the liquid-like characteristics (δ_loss_) of cell viscoelasticity (since in local measurement, the surface tension adds effectively to the ratio of the real part to the imaginary part of the complex modulus). Our result constitutes the first unambiguous measurement of the elastocapillary effect in AFM indentation of live cells, underscoring the crucial role of surface tension in the mechanical response of the cell. The nanodiamond quantum sensor is also demonstrated as a useful tool for quantifying the intrinsic mechanical properties of soft and complex biological systems.

## Results and Discussion

### Dynamic Nonlocal Deformation Measurement Based on ND Rotation Sensing

**Figure 1:**
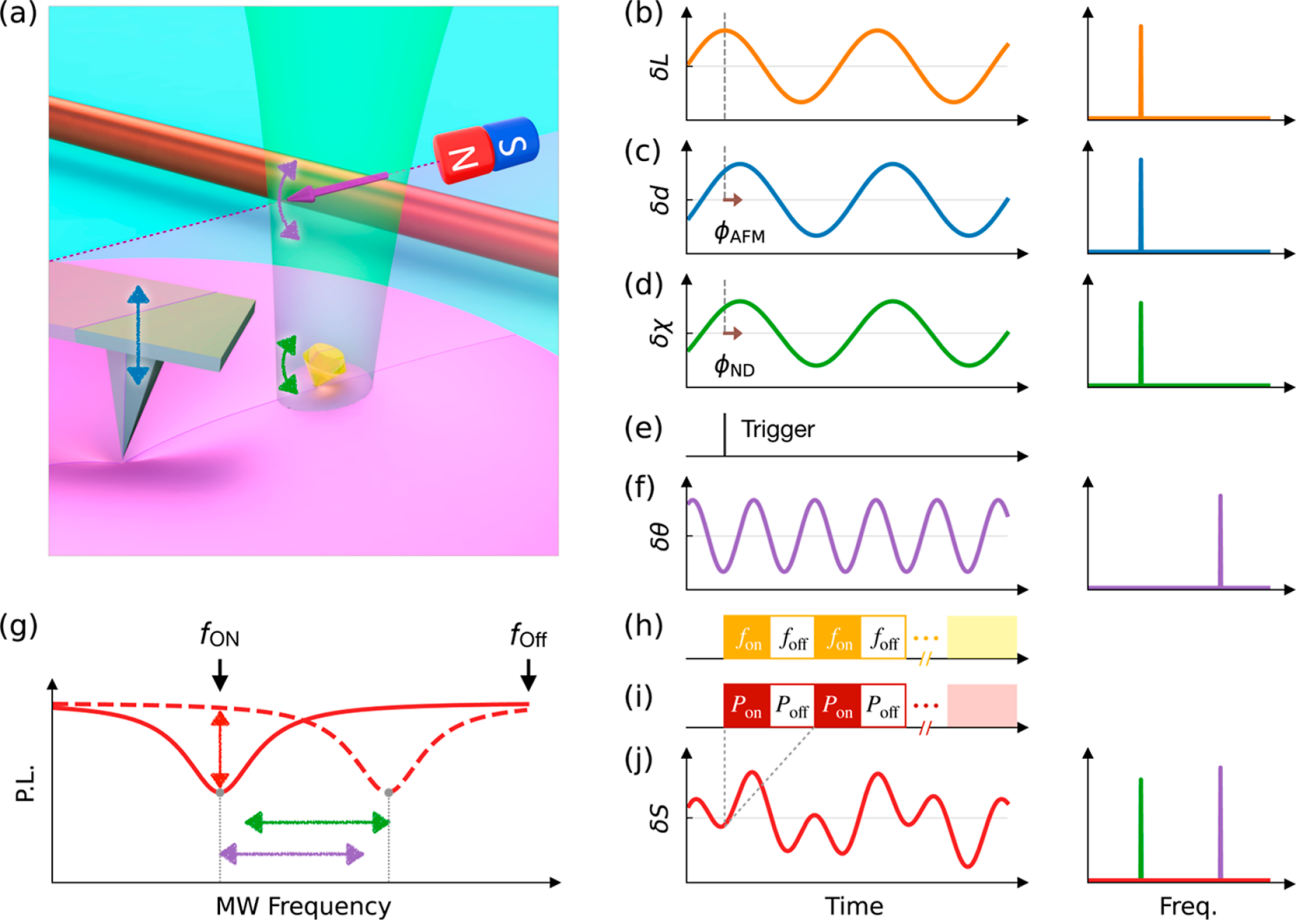
Dynamic nonlocal deformation reconstruction using nanodiamond (ND) rotation sensing. (a) Schematic of an atomic force microscope (AFM) tip imposing an oscillatory indentation on a soft material. NDs are attached on the surface for dynamic rotation sensing by optically detected magnetic resonance (ODMR). (b–d,f), Time evolution (left) and the corresponding spectra (right) of the loading *L*, indentation depth *d*, ND rotation angle χ, and magnetic field rotation angle θ. The brown arrows in (c,d) indicate the local and nonlocal phase lags (ϕ_AFM_ and ϕ_ND_). (e) Schematic of the trigger signal sent by the AFM for synchronizing with the ODMR measurement. (g) Schematic of the two-point ODMR method. The red lines show the changes of ODMR spectra with consequent oscillations in the emission of the ND (indicated by red arrow) due to the rotation of ND (indicated by green arrow) and that of magnetic field (indicated by purple arrow). The on- and off-resonance frequencies (*f*_on_ and *f*_off_) of the nitrogen-vacancy (NV) centers spin transitions are indicated by the black arrows. (h,i) Alternation of the microwave frequencies and the corresponding fluorescence counting for each data of the two-point ODMR signal. (j) Time evolution (left) and the corresponding spectrum (right) of the two-point ODMR signal *S*. The corresponding peaks in the spectrum induced by the oscillatory loading and the rotating magnetic field are colored by green and purple, respectively.

Figure 1 illustrates the scheme for the dynamic nonlocal deformation measurement. We used a home-built AFM-confocal correlated microscopy setup (for details, see Methods). We applied shallow indentations (500–800 nm indentation depth) to soft materials [see Figure 1a]. Upon a certain holding force, we added a modulation [Figure 1b] to induce an oscillatory indentation at a fixed frequency [Figure 1c]. The oscillatory indentation, through surface deformations, caused oscillatory rotations of the NDs preattached on the sample surfaces [Figure 1d]. Through phase-sensitive spectral analysis of the ND rotations, we isolated the indentation-induced deformation signals, from those originating from fluctuating environmental parameters and/or cellular activities (in the case of live cells).

For the phase-sensitive detection of dynamic ND rotation, two required techniques are developed here. First, an accurate synchronization between the force loading and the nonlocal deformation measurement was achieved by using a trigger signal to align the start of data recording [Figure 1e], which is important for precise measurement of the phase lag between force and deformation. Second, we developed a fast rotation sensing scheme based on a two-point ODMR method with high duty ratio, to overcome the limit of time resolution (in the order of seconds) of the previous ND rotation measurement using continuous-wave ODMR,^20^ which is inadequate for tracking the dynamic nonlocal deformation of the sample. The method is enabled by applying an external magnetic field with a known oscillatory rotation. This magnetic field serves as a reference for calibrating the rotation of the ND, eliminating the need to record the full ODMR spectra to obtain a complete set of rotation information.^10^ Such calibration is realized by the dependence of the ground state spin resonance frequency of NV centers (*f*_±_) on the magnetic field components along the NV axis with *f*_±_ ≈ *D* ± γ_e_**B**(*t*) × ***n***_NV_(*t*), where *D* is the zero-field splitting and γ_e_ is the electron gyromagnetic ratio.^23,24^ Changing the orientation of ND [represented by ***n***_NV_(*t*)] and that of the magnetic field [represented by **B**(*t*)] has equivalent effects on the resonance frequency *f*_±_. Assuming a known rotation axis of the ND upon indentation,^10,11^ we applied a known rotating magnetic field with the same rotation axis but a different oscillating frequency [refer to Figure 1d,f]. This allows us to determine the magnitude of the ND rotation caused by indentation from the magnitude of the magnetic field rotation. The details of the magnetic field control and calibration are given in Methods.

In the two-point ODMR method, as depicted in Figure 1g, the photon emission is collected at an on-resonance microwave (MW) frequency and an off-resonance frequency to normalize fluorescence fluctuation (see Methods for data analysis). The pulse sequences of microwave control and fluorescence acquisition are given in Figure 1h,i. The coaxial oscillatory rotation of the ND and that of the magnetic field induces a peak shift of ODMR and consequent oscillations in the on-resonance emission of the ND. Figure 1j (left) illustrates the normalized ODMR signal of the ND as a function of time, displaying a beat signal resulting from the two frequency components. The spectrum obtained by Fourier transformation in Figure 1j (right) exhibits two distinct peaks corresponding to the rotation of the ND and the magnetic field. Since the rotating magnetic field is precalibrated and known, both the amplitude and phase of the ND rotation can be determined from these peaks. The complete measurement sequence of nonlocal deformation sensing is given in Supporting Information Figure S2.

To further enhance the signal-to-noise ratio of the measurement, we not only utilize one resonance peak in ODMR but also simultaneously incorporate the four resonance peaks originating from the four NV orientation classes in the ND by applying a mixture of the four on-resonance MWs in the ODMR detection process (see Supporting Information Figure S5 for details). This approach enhances the contrast of the two-point ODMR measurement by a factor of approximately four.

### Proof-of-concept Demonstration: the Spatial–Temporal Mechanical Response of PDMS

**Figure 2:**
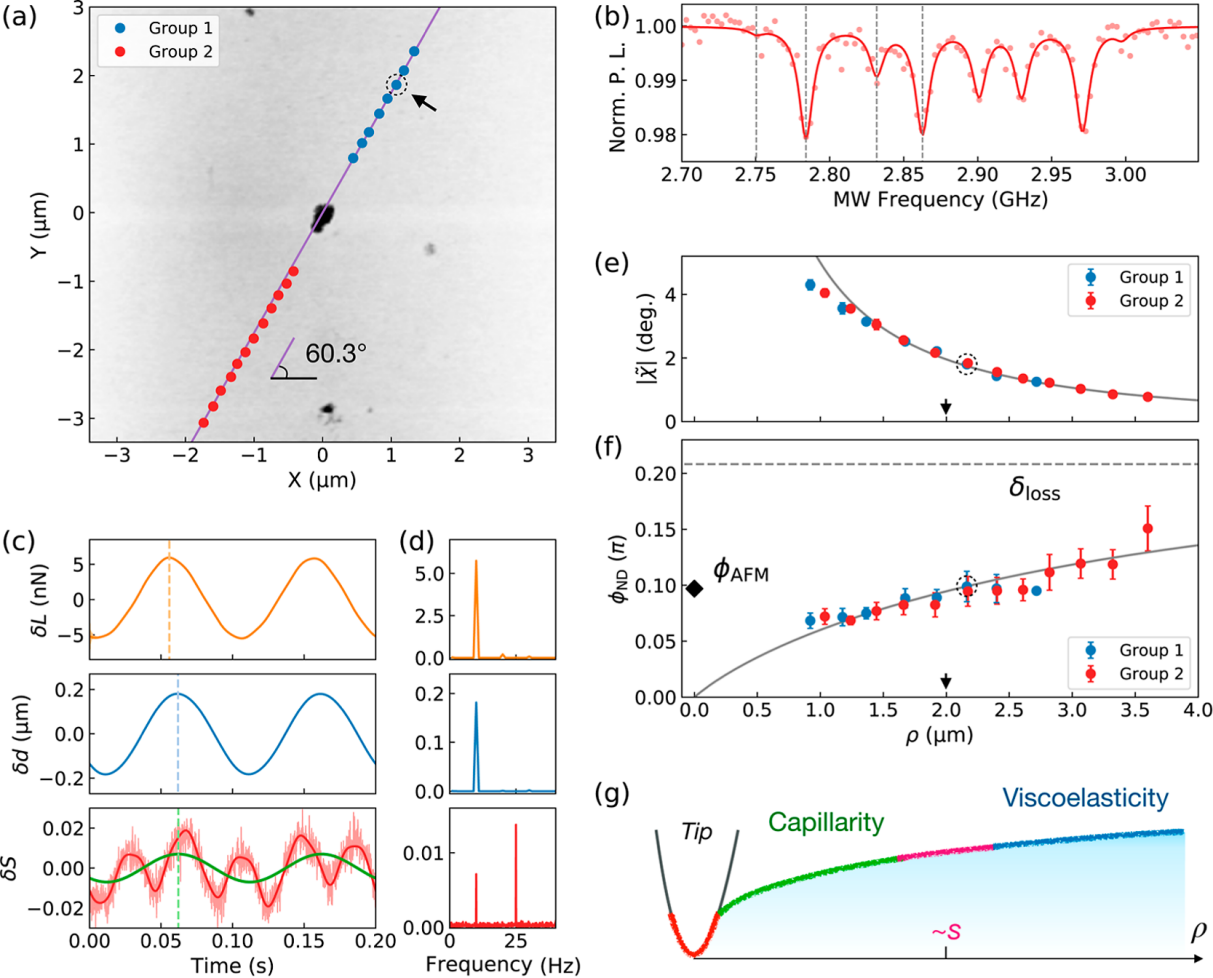
Dynamic nonlocal deformation measurement using ND rotation sensing on polydimethylsiloxane (PDMS). (a) AFM image of a typical PDMS surface with an ND located at the center (the origin). The blue and red dots represent the indentation locations of the AFM tip on the upper right (Group 1) and the lower left (Group 2) of the ND. The purple line indicates the direction of the external magnetic field B (60.3° from the *x*-axis). (b) ODMR spectra of the ND under the magnetic field obtained before the indentations. The four resonance frequencies are indicated by the gray dashed lines. (c) Time-dependent data and (d) Fourier transform (FT) of the loading *L*, the depth *d* of the AFM tip, and the two-point ODMR signal *S* at the indentation location indicated by the black arrow in (a). The red solid curve is the simulation of the beat signal based on the deduced amplitude and phase, while the green line is the extracted 10 Hz signal of ND rotation induced by indentation. The dashed colored lines in (c) show the phase shifts of the local depth and the extracted nonlocal ND rotation relative to the loading. (e) Amplitude () and (f) the nonlocal phase lag (ϕ_ND_) of the oscillatory rotation angle as functions of the distance ρ between the indentation location and the ND. The data points are averages over 10 repeated measurements executed at each location. The fitting results of the linear viscoelastic model with surface tension effect are plotted by the gray lines, where the elastocapillary length |s̃| is pointed by the black arrows in (e,). The local phase lag ϕ_AFM_ deduced from the AFM data is drawn by the rhombus at ρ = 0, while the bulk loss angle δ_loss_ is indicated by the gray dashed line. The error bars in (e,f) are the standard derivation of the repeated measurements. (g) Schematic of the elastocapillary effect in AFM indentation.

As a proof-of-concept demonstration, we conducted measurements on the dynamic nonlocal deformation of a PDMS film (see the Methods for sample preparation). Figure 2a presents the AFM topography image of a PDMS film with NDs anchored on its surface. We performed sequential AFM indentations at 20 different locations near an ND (see Supporting Information Figure S6 for more information). The locations were divided into two groups on two sides of the ND sensor, and the displacements from the AFM indentation spots to the ND were set parallel to the projection of the external magnetic field on the *xy*-plane (see the Methods for more information). This alignment ensured that the magnetic field and ND shared the same rotation axis as the deformation is axisymmetric under the loading by an axisymmetric tip.^10,11^

Prior to the indentations, we recorded an ODMR spectrum to determine the resonance frequencies, as shown in Figure 2b. For each measurement, synchronized AFM oscillatory indentation and two-point ODMR collection were performed. Figure 2c illustrates one set of AFM and ODMR data as a function of time. Detailed information about the corrections for laser reflection and hydrodynamic drag on the AFM cantilever can be found in the Methods section. The corresponding data in the frequency domain, obtained through Fourier transformation (FT), are presented in Figure 2d. The top two panels of Figures 2c,d display the local indentation data, including loading and indentation depth, obtained from AFM measurements. The bottom panel depicts the two-point ODMR signal of the ND, revealing the beat of two frequency components at 10 and 25 Hz, corresponding to the loading and magnetic field modulation, respectively. The amplitude and phase of each oscillation were derived from the FT signal at the corresponding frequency (see Methods for details on data analysis). By comparing the phases, we obtained the phase lag of the local deformation ϕ_AFM_ and nonlocal deformation ϕ_ND_ relative to the loading.

Figures 2e,f display the oscillation amplitude  of the ND rotation angle and the phase lag ϕ_ND_ between loading and nonlocal deformation at various distances ρ between the indentation positions and the ND. The two sets of data, obtained from the two sides of the ND [see Figure 2a], exhibit good agreement, underscoring the consistency of the measurements on the homogeneous sample. The rotation amplitude decreases with increasing distance ρ, while the phase lag increases with the distance. This spatially dependent phase lag indicates the interplay between capillarity (characterized by the time-independent surface tension τ_0_ at the material–liquid interface) and viscoelasticity (characterized by the frequency-dependent complex modulus  of the material). This interplay becomes significant when measured at a lateral length scale comparable to the elastocapillary length , as depicted in Figure 2g.^12^ In the regime where , the time-dependent bulk viscoelasticity dominates, with a phase lag determined by the material’s loss angle δ_loss_. Conversely, in the deformation regime where , the time-independent capillary force dominates, resulting in no phase lag.

According to Saint-Venant’s principle,^1^ the nonlocal deformation far away from the indentation point (with distance ρ > 800 nm, much larger than the tip radius of approximately 25 nm) is independent of the local factors of the AFM indentation and can be well approximated by the deformation upon point loading.^13^ Therefore, a linear viscoelastic model based on a point loading accounting for the surface tension effect^13,25^ is used to explain the observed nonlocal mechanical response of the PDMS (see Methods for details of the model). The viscoelastic properties and surface tension of the PDMS, measured at 10 Hz, are deduced to be , δ_loss_ = 0.21(2)π, and τ_0_ = 7.4(6) mN m^–1^, which are consistent with previous reports.^16,26^ The results indicate an elastocapillary length of , as indicated by the black arrows in Figure 2e,f. For comparison, the derived δ_loss_ of the PDMS is plotted in Figure 2f with a gray dashed line, and the local phase ϕ_AFM_ obtained from conventional AFM dynamic measurements is represented by the black rhombus at ρ = 0. The local phase at ρ = 0 deviates from the fitting results of zero phase lag (when only the static surface tension is considered). This deviation is induced by the local factors of the AFM indentation, where the point-loading approximation is invalid.

**Figure 3:**
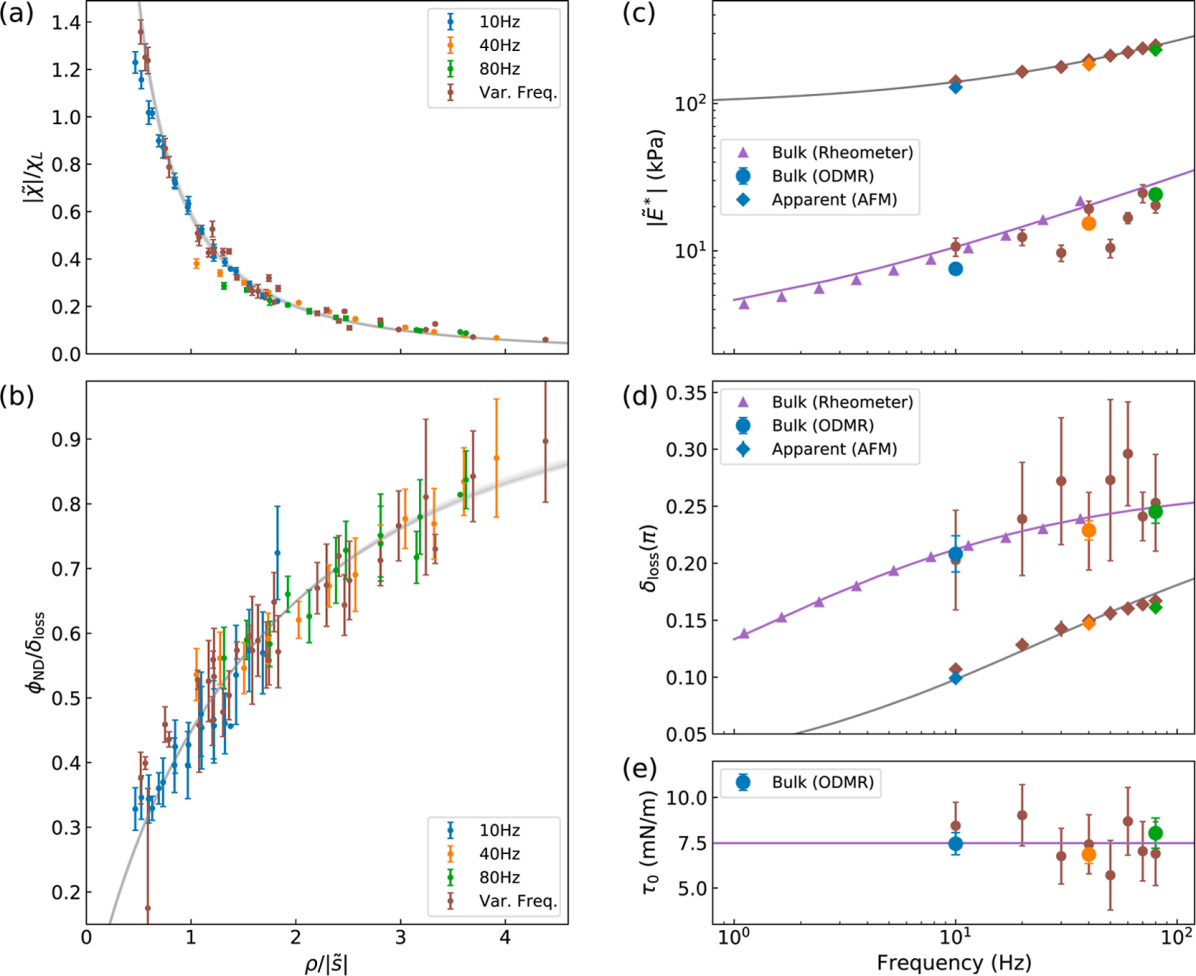
Evaluation of the complex modulus and surface tension of the PDMS film. (a) Rescaled amplitude of the oscillatory rotation angle () and (b) normalized nonlocal phase lag (ϕ_ND_/δ_loss_) of the oscillatory rotation angle as functions of the rescaled distance  between the indentation spot and the ND. The simulation results of the linear viscoelastic model with surface tension effect are plotted by the gray lines. The error bars in (a,b) are similar to those of Figure 2e,f. (c) Evaluated magnitude  and (d) loss angle δ_loss_ of the complex modulus as functions of frequency. The purple triangles (colored rhombuses) plot the magnitude and loss angle of the bulk (apparent) complex modulus of PDMS measured by rheometer (AFM). The lines are the fitting results of the bulk and apparent elastic moduli by using the power-law function as illustrated in the main text. (e) Surface tension τ_0_ deduced in the different experiments. The line indicates the mean value. The error bars in (c–e) are fitting errors.

To further demonstrate the method’s capability in evaluating the frequency-dependent viscoelasticity, the nonlocal deformation measurements were repeated using different loading modulation frequencies ranging from 10 to 80 Hz (for detailed information, refer to Supporting Information Figures S6, S10, and S11). In Figure 3a,b, the amplitude of ND rotation, normalized by the constant , and the nonlocal phase, normalized by δ_loss_, are plotted as functions of the rescaled distance ρ/. The parameters χ_L_ and δ_loss_ are obtained by fitting the nonlocal deformation data by using the point-loading model with elastocapillary effects, and the fitting results are represented by the gray lines. The rescaled results obtained at different frequencies approximately align along the same line, following the approximately universal function provided by the viscoelastic model with capillary effects (see Methods). The standard deviation of the oscillation amplitude  was , which defines a nanometer precision of deformation reconstruction in the loading direction^10^ (see Supporting Information Figure S12). Meanwhile, the standard deviation of the phase lag ϕ_ND_ was , which defines a tens of microsecond precision of time-lag measurement (63 us for the 80 Hz oscillation).

The mechanical properties of PDMS at different frequencies were deduced by fitting the nonlocal deformation data corresponding to the respective frequencies. The deduced complex modulus (, δ_loss_) and surface tension (τ_0_) are shown as functions of the loading modulation frequency, represented by colored circles in Figure 3c–e. Both the modulus  and the loss angle δ_loss_ increase with frequency, while the surface tension exhibits no apparent frequency dependence with a mean value of 7.5 mN m^–1^, which is in agreement with a recent report.^16^ The bulk viscoelastic properties of the PDMS were also measured with a rheometer (see Methods), represented by the purple triangles in Figure 3c,d. As PDMS exhibits scale invariance as cross-linked polymer networks, the bulk complex moduli were fitted using a power-law function , with the static modulus *E*_0_^*^ = 2 kPa, the characteristic time τ = 0.08 s, and the exponent *n* = 0.55. The mechanical properties evaluated from the dynamic nonlocal deformation measurements are consistent with the results from the rheometer and also with the literature.^26^

For comparison, the apparent complex modulus  was calculated using the local dynamic loading and deformation data (refer to Supporting Information Figure S13) by the conventional Hertz-Sneddon model,^27,28^ with the surface tension effect neglected (see Methods). The apparent loss angle δ_apparent_ by the conventional AFM method was obtained from the measured local phase lag of the indentation depth relative to the loading, i.e., ϕ_AFM_. The apparent modulus  and the loss angle δ_apparent_ are represented by solid rhombuses in Figure 3c,d, respectively, which deviate significantly from the bulk viscoelastic properties obtained through nonlocal measurements and oscillatory rheology (i.e.,  and . These deviations suggest that neglecting the surface tension in conventional methods would lead to an overestimation of the apparent modulus [Figure 3c] and an underestimation of the loss angle [Figure 3d]. The same measurements were repeated on additional PDMS samples, yielding similar extracted mechanical properties, as shown in Supporting Information Figure S14.

### Spatial–Temporal Mechanical Response of Live Cells

**Figure 4:**
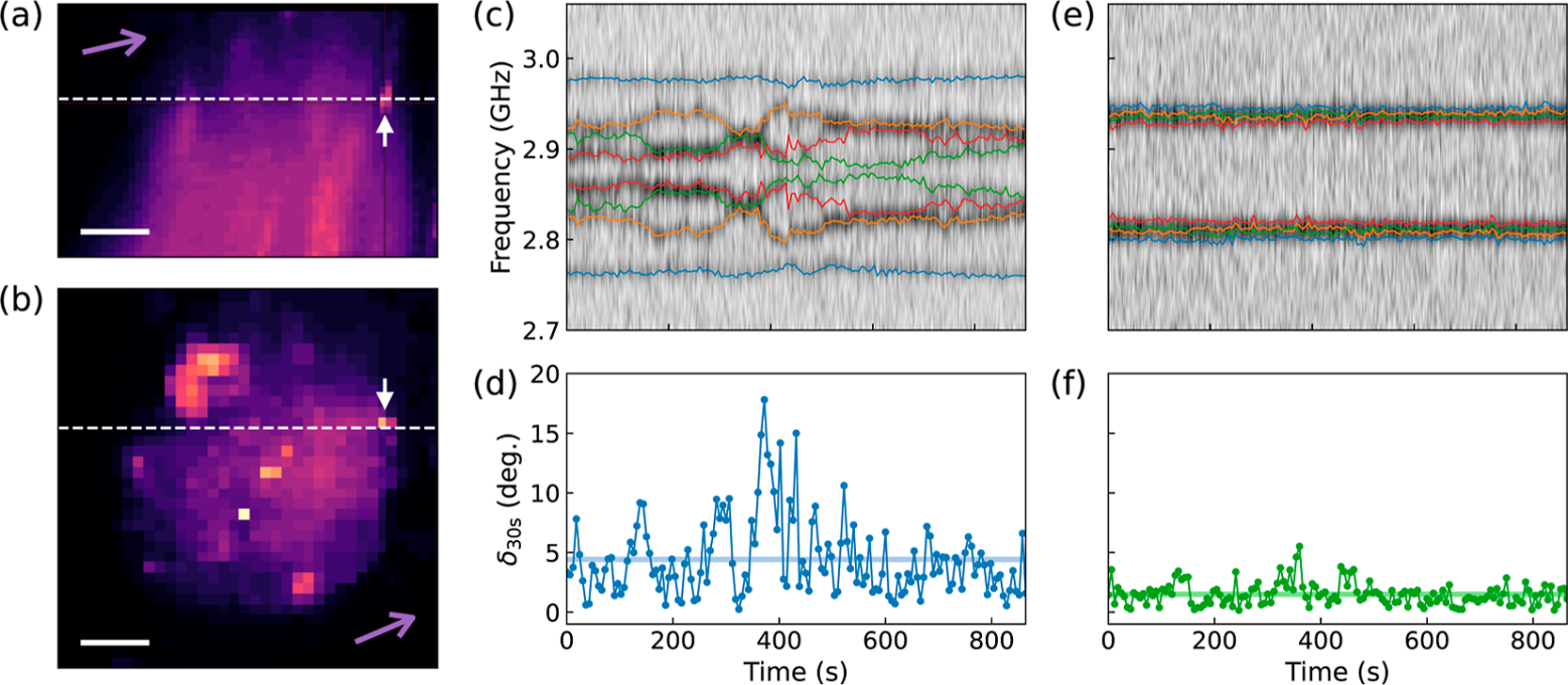
ND rotation caused by live cell activity. (a) *xz*-cross section and (b) *xy*-cross section of the confocal fluorescence images of the MCF-7 cell with an ND on the cell membrane as indicated by the white arrows. The white dashed lines show the cutting positions of the cross sections. The scale bars are 5 μm. The purple arrows indicate the projected directions of the external static magnetic field, showing that the direction of the magnetic field is approximately normal to the cell surface plane where the ND attached. (c) Time-dependent ODMR spectra collected from the ND on live MCF-7 cell. The fitting results of the resonant frequencies are overlapped on the raw data as colored lines. The contrast of the ODMR spectra is encoded in greyscale. (d) Out-of-plane rotation angle δ_30s_ of the ND within 30 s time duration. The blue line is the mean value. (e,f) are similar to (c,d) but which are the results measured after the cell died.

Live cells are complex active materials exhibiting both solid-like elastic and liquid-like viscous features. The activities of such live biological samples could induce random rotation^20^ of NDs attached on the sample surface even in the absence of external forces. We evaluated the typical behaviors of such rotation noises by measuring the time-dependent ODMR spectra of single NDs anchored to the plasma membranes of live cells (see Methods for cell culture, cell status, and sample preparation). Figures 4a,b present side and top views, respectively, of the confocal fluorescence images of an MCF-7 cell. The ODMR spectra were collected under an external magnetic field applied approximately normal to the cell surface plane where the ND was attached. The out-of-plane rotation of the ND (with the rotation axis on the plane, the same form of the rotation induced by deformation) can be extracted from the ODMR (see Methods). Figure 4c shows the time-dependent ODMR spectra overlapping with the fitting results of the resonance frequencies (the colored lines) obtained on live MCF-7 cells. The extracted out-of-plane rotation angle of the ND within 30 s duration is presented in Figure 4d, showing a mean value of 4.4° of the rotation angle and revealing the cell activities. As a comparison, the ODMR spectrum measured after the cell died [Figure 4e] shows less rotation with a mean value of 1.5° [Figure 4f]. The rotation could be induced by other noise sources such as rotation diffusion, shot noise, and other perturbations. The results indicate that the typical rotation noise caused by cell activities and rotation diffusion is comparable to the rotation signal induced by AFM indentation on soft samples [typically in the order of degrees, see Figure 2e]. This could lead to the submerging of signal from noise in the static nonlocal deformation sensing method used before.^11^

**Figure 5:**
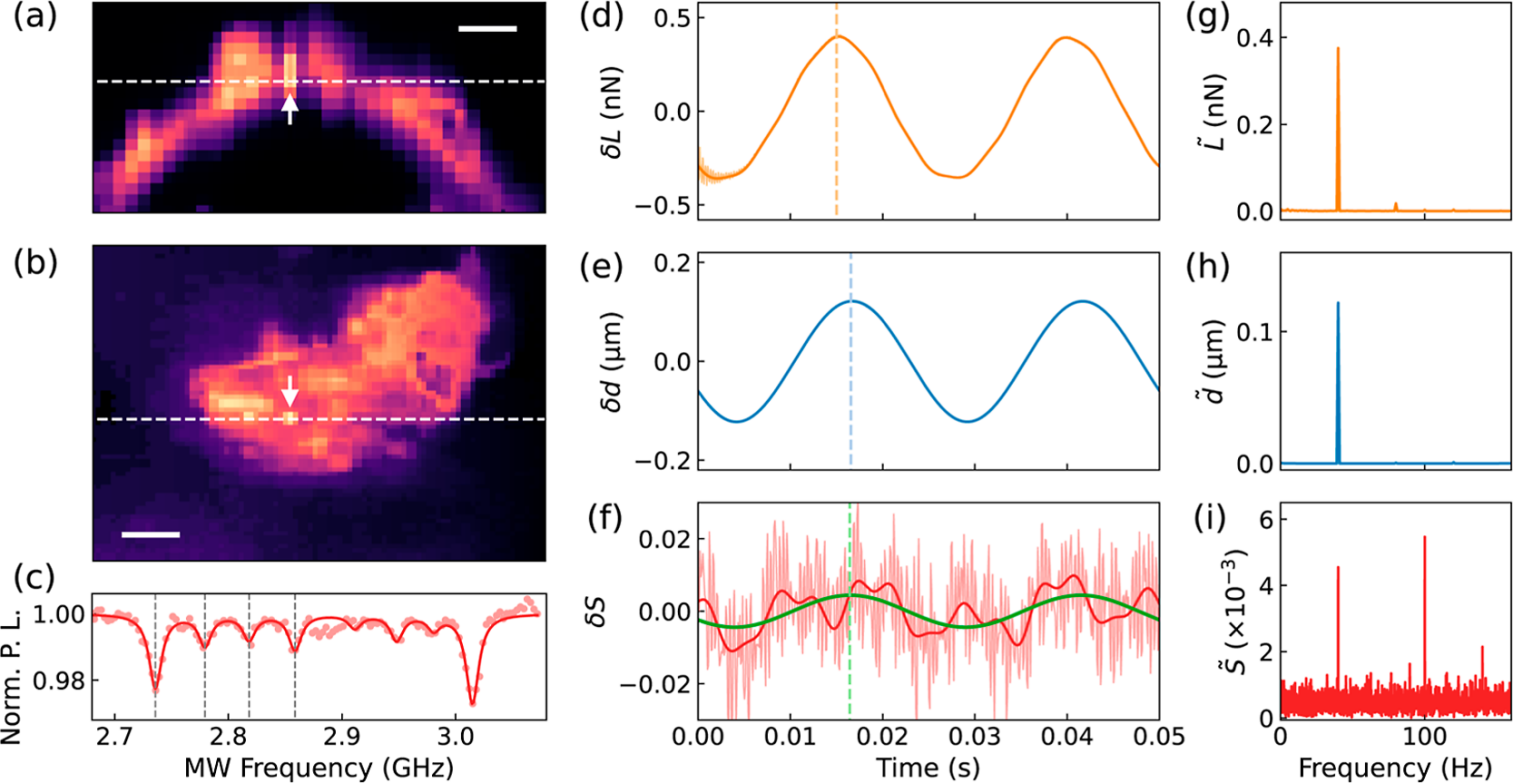
Typical AFM and ODMR data of the nonlocal deformation measurement on live cells. (a) *xz*-cross section and (b) *xy*-cross section of the confocal fluorescence images of the MCF-7 cell with an ND on the cell membrane as indicated by the white arrows. The white dashed lines show the cutting positions of the cross sections. The scale bars are 5 μm. (c) Typical ODMR spectra of the ND obtained before the indentations. The gray dashed lines indicate the four resonance frequencies. (d–f) Time-dependent data and (g–i) Fourier transform (FT) of the loading *L*, the depth *d* of the AFM tip, and the two-point ODMR signal *S*. The red solid curve is the beat signal simulated using the deduced amplitude and phase. The green line is the extracted 40 Hz signal of the indentation-induced rotation of ND. The dashed colored lines in (d–f) show the phase shifts of the depth and the ND rotation relative to the loading.

Here, we applied our dynamic nonlocal deformation sensing method to obtain the spatial–temporal mechanical response of live cells. We measured the rotation of a single ND on live MCF-7 cells upon AFM indentation. Figures 5a,b present the confocal fluorescence images of the MCF-7 cell with an ND indicated by the white arrows. Although the orientation of the ND changed over time [Figure 4c], we collected the ODMR spectrum before each indentation to obtain the corresponding four transition frequencies [Figure 5c] and then performed the AFM indentation (with a modulation frequency of 40 Hz) and two-point ODMR measurements. Figures 5d–f illustrate one set of the loading, the indentation depth, and the two-point ODMR data as functions of time. The corresponding FT results are shown in Figure 5g–i. The two peaks in the FT result of the ODMR signal reveal the oscillatory rotation of the ND (40 Hz) with an amplitude of 3.3° ± 0.2°. The result shows that through the frequency-domain measurement, the rotation signal with an amplitude of several degrees can be singled out from the background rotation noise of the same order of magnitude (originated from cell activities, rotation diffusion, and other perturbations from environments). The method enhances the signal-to-noise ratio of the measurement and enables the measurement of the spatial–temporal mechanical response of live cells upon AFM indentation.

**Figure 6:**
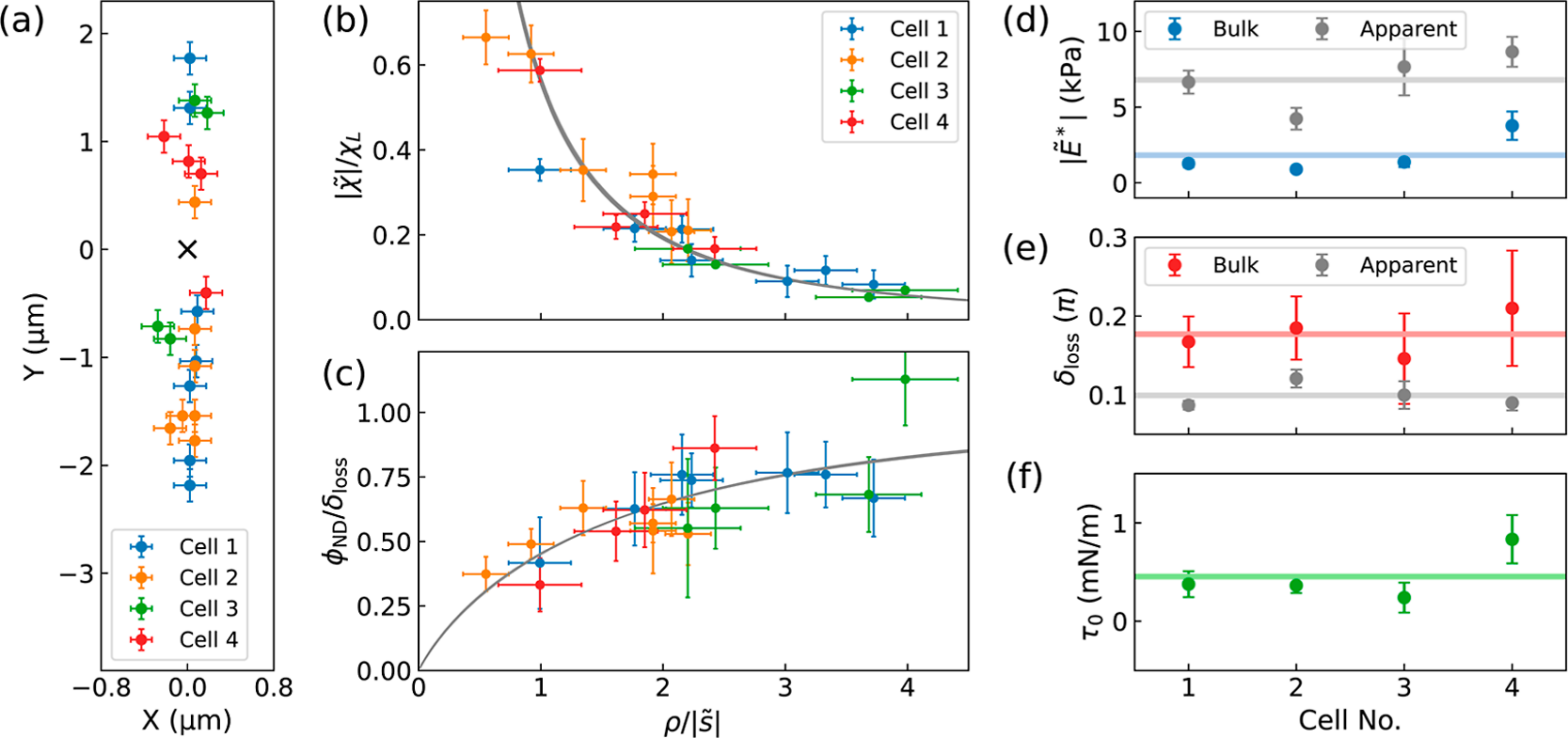
Evaluation of the complex modulus and surface tension of MCF-7 cells. (a) Displacements of the indentation spots from the NDs (denoted by the black cross at the origin). The AFM indentations were performed along the *y*-axis, parallel to the direction of the external magnetic field. (b) Rescaled amplitude () and (c) normalized phase lag (ϕ_ND_/δ_loss_) as functions of the rescaled distance . The simulation results are plotted by the gray lines. The error bars in (a) and *x*-axis error bars in (b,c) are the optical resolution (±150 nm). The *y*-axis error bars in (b,c) are estimated form the signal-to-noise ratio of the FT data of the two-point ODMR signal. (d) Evaluated magnitude  and (e) loss angle δ_loss_ of the bulk (colored dots) and apparent (gray dots) complex modulus of the four live cells. (f) Surface tension τ_0_ of the four cells. The lines in (d–f) indicate the mean value. The error bars in (d–f) are fitting errors.

The measurements were performed at different positions near the ND, as shown in Figure 6a. The normalized amplitude and the nonlocal phase of ND rotation upon AFM indentation are plotted as functions of the rescaled distance in Figure 6b,c. The data are well fitted by a viscoelastic model that includes the surface tension effect, allowing estimation of the mechanical properties of the MCF-7 cell as , δ_loss_ = 0.17(3)π, and τ_0_ = 0.4(1) mN m^–1^. Similar measurements were conducted on three additional MCF-7 cells, and their indentation positions and nonlocal deformation data are also presented in Figure 6a–c. The normalized data from all cells were well described by the viscoelastic model with the inclusion of the surface tension. The observed spatially nonuniform phase of nonlocal deformation suggests that the surface tension significantly influences the mechanical response of live cells during AFM shallow indentation. Figures 6d–f display the deduced complex modulus ( and δ_loss_) and surface tension (τ_0_) of the four live cells based on the ND rotation data. The deduced magnitude and loss angle of the complex modulus are in the range of 1–4 kPa and 0.14–0.22π at 40 Hz oscillation frequency, which are consistent with the previous reports measured by AFM (on the order of kPa and 0.1–0.2π at frequencies of several tens of Hz).^5,29,30^ The deduced surface tension (on the order of 10^–1^ mN m^–1^) agrees with the range of the membrane tension and cortex tension measured in the literature.^31,32^

As a comparison, the apparent complex moduli () of the cells were evaluated from the local deformation data (see Supporting Information Figure S16) using the Hertz-Sneddon model, represented by gray dots in Figure 6d,e. Considering surface tension in the contact model is also found to be crucial for accurately assessing cell mechanical properties, similar to the previously discussed results obtained from PDMS. Neglecting surface tension, as done in conventional AFM indentation methods, would lead to an overestimation of the apparent modulus of the cell and an underestimation of the loss angle, indicating an underestimation of the liquid-like characteristics of the cell.

## Conclusions

In summary, we have developed a dynamic nonlocal deformation sensing scheme for spatial–temporal mechanical analysis using modulated AFM nanoindentation and frequency-specific ND rotation sensing. We successfully demonstrated frequency-dependent dynamic nonlocal deformation mapping with high precision and high spatial and temporal resolution on soft viscoelastic PDMS and live cells. The measurements on cells demonstrate the robustness of the method against background deformation noise caused by cellular activities. Our method leads to the first measurement of the spatially dependent phase lagging of nonlocal deformation, resulting from the interplay between viscoelasticity and capillarity. Importantly, our nonlocal measurement approach allows for an unambiguous quantitative analysis of the intrinsic mechanical properties of soft materials, including the viscoelastic complex modulus and surface tension. Our results highlighted the elastocapillary effect at the material/liquid interface of viscoelastic materials, such as polymers and live cells, during AFM indentation. We showed that neglecting the surface tension effect in contact mechanics leads to an overestimation of the apparent complex modulus and an underestimation of the loss angle of the materials.

The important insights into the competition between viscoelasticity and capillarity bring new inspiration to studies of cell mechanics. While the surface tension of live cells^32^ (with a typical value of 10^–1^ mN m^–1^) does not change with frequency, the elastic modulus of cells increases from 10^–2^ to 10^1^ kPa in the frequency range of 10^–1^ to 10^5^ Hz^5^. Consequently, the elastocapillary length of the cells would change significantly from tens of micrometers to tens of nanometers. Since the deformation length scale is frequency-independent (typically 10^–1^ to 10 μm), the large variation in the elastocapillary length at different frequencies could cause bias on the evaluation of the frequency-dependent rheological characteristics of cells, especially in the low frequency regime. By considering the surface tension effect, it would be interesting to revisit some previous discussions on cell mechanics based on the local measurement of apparent elastic modulus,^5,33^ for example, the explanations of the weak power-law rheological behaviors of cell at low frequencies.^34^ Overall, the nanodiamond-based dynamic nonlocal deformation sensing offers a unique tool and opens up new possibilities for studying the mechanics of live cells and other soft biorelevant viscoelastic materials, with potential applications ranging from investigating cell rheological behaviors to establishing cancer diagnostic mechanical fingerprints.

## Methods

### AFM-Confocal Correlated Microscopy

The measurements were carried out on a home-built confocal-AFM correlation microscope (see Supporting Information Figure S1 and also Supplementary Note S1 of ref (10) for more details). We used a laser scanning confocal system for imaging and ODMR measurements. The spins of NV centers were pumped by a 532 nm laser and manipulated by microwave delivery via a copper wire antenna (20 μm diameter). A BioScope Resolve AFM (Bruker) instrument was used for surface imaging and for nanoindentation.

#### Spatial Correlation

For the PDMS experiments, the spatial correlation between the atomic force microscope and the confocal microscope was established by overlapping the AFM image and the fluorescence image of NDs on the samples.^10^ The AFM imaging provides the coordinates of different indentation spots with spatial resolution limited by the tip radius (∼25 nm, DNP-10-A, Bruker). For the live cells experiments, the position of the AFM tip in the confocal image was determined by the same method as above, but the overlap of the images was performed on NDs on the substrate (cover slide).^10^ Then, the displacement of the ND from the indentation position was read from the confocal image with an optical resolution (∼300 nm).

#### Synchronization

In the AFM-confocal correlation microscope, we used NIDAQ (PCIe-6363, National Instrument) for counting ND fluorescence and High Speed Data Capture (HSDC) (Nanoscope, Bruker) for AFM data acquisition, both with time resolution of 50 μs. The fluorescence and AFM data collections in the two microscopies were triggered by the same pulse signal sent out at the start of the force modulation (a negative-going pulse with width of 20 μs from the NanoScope V Controller of the AFM microscope; see Supporting Information Figure S2 for the time sequences of the measurements). The synchronization of the two data collections were checked by collecting the fluorescence variation of an ND attached on the AFM cantilever under a noncontact height modulation of the AFM tip (with the tip height acquired by AFM), as shown in Supporting Information Figure S3. The lagging time between the two sets of data is less than 60 μs.

### Oscillatory Rotation of the Magnetic Field

The coaxial oscillatory rotation of the magnetic field was realized by applying a static magnetic field nearly in the *xy*-plane using a pair of permanent magnets and a controllable magnetic field in the *z* direction by a magnetic coil with adjustable current (ranging between ±0.5 A), see Supporting Information Figure S4a for details. The oscillation of the magnetic field in the *z* direction, realized by applying AC current to the magnetic coil, causes a rotation of the resultant external magnetic field applied on the ND, where the rotation axis is perpendicular to both the static magnetic field and the *z*-axis [see Supporting Information Figure S4b].

Before each set of indentation experiments, the external magnetic fields were calibrated using the ODMR spectra of the NV centers in a bulk diamond crystal. Two examples of the calibration are shown in Supporting Information Figure S4c–g. The rotation angle of the magnetic field, induced by the AC current in the magnetic coil, had slopes of 19.4 and 13.4 deg./A in the PDMS and the live cell experiments, respectively [see Supporting Information Figure S4g]. The variation of the magnitude of the external magnetic field during modulation (±0.2 A) is less than 2% and is neglected [see Supporting Information Figure S4e]. The polar angles of the projection of magnetic field on the *xy*-plane are 60.6 and 90.2 deg in the PDMS and live cell experiments, respectively.

### PDMS Sample Preparation

PDMS films were prepared using a Sylgard 184 elastomer kit (Dow Corning). The silicone base and cross-linker were thoroughly mixed with a weight ratio of 60:1 before degassing under vacuum. For AFM indentation experiments, the samples were prepared by spin-coating the mixture at 3000 rpm for 30 s onto a cover glass and cured at 60 °C for 24 h. The thicknesses of the films are typically ∼40 μm, measured by confocal microscopy. The roughness of the PDMS surface is below 6 nm, measured by AFM. 20 μL aqueous solution of ND with a concentration of 2 μg/mL (from Adámas Nanotechnologies, ND with ∼900 NVs and diameter of 140 nm) was then drop-casted onto the PDMS film. The cover glass with the PDMS film was glued to a PCB board, followed by soldering a microwave antenna (a copper wire with 20 μm diameter) on the surface of the sample connecting the microwave transmission lines on the PCB board. A confocal dish was then glued to the cover glass to form a liquid chamber, with water added before experiments.

### Cell Culture and Incubation with NDs

MCF-7 cells were seeded in a confocal dish and incubated at 37 °C with the CO_2_ level (5%) and humidity controlled. The dish was prepared in advance with a microwave antenna bonded to the surface of the cover glass. The growth medium of the MCF-7 cell was Dulbecco’s modified Eagle’s medium (DMEM, Gibco) supplemented with 10% fetal bovine serum (FBS, Gibco) and 1% penicillin–streptomycin (Gibco).

Cells were cultured for 48 h before introducing NDs. The NDs (from Adámas Nanotechnologies, ND with ∼900 NVs and diameter of 140 nm) were dispersed in sucrose solution (5 μg mL^–1^ concentration) and incubated with cells for 20 min at 37 °C in the incubator. With NDs attached on the cell membrane after incubation, the sample was washed with PBS three times and supplied with fresh DMEM-FBS medium. The dish was mounted onto the stage of AFM, with 5% CO_2_ supplied during the indentation experiments. For imaging of cells by confocal microscopy, the plasma membrane of cells was labeled by the CellMask Green Plasma Membrane Stain (Thermo Fisher).

Before applying the nonlocal deformation sensing technique, we evaluated the effect of ODMR measurements on cell viability. Under the typical experimental condition (the concentration of nanodiamond solution, the microwave, and laser power) of the AFM experiments, we used the viability assay BCECF AM (Thermo Fisher) to assess cell viability (see Supporting Information Figure S18). The result shows no significant change in cell viability within 2 h, which is longer than the typical duration of a group of deformation sensing experiments. Cell viability shows reduction under longer measurement time (>6 h), which may be caused by laser phototoxicity.

During the indentation experiments on cells, the complete spectrum ODMR was collected before each indentation. The ODMR spectra were observed to change with time in contrast to those obtained on dead cells (see Figure 4 and Supporting Information Figure S17), suggesting that the cell sample remained alive during the experiment.

### AFM Nanoindentation

A DNP-10-A/PFQNM-LC-A tip was used in the experiments measuring the nonlocal deformation of PDMS/live cells upon AFM indentation (AFM: BioScope Resolve, Bruker). For a laterally homogeneous sample, its deformation resulting from an indentation at a fixed position can be effectively assessed by measuring the rotation of a single ND during multiple indentations conducted around the ND.^10^ Consequently, we performed sequential AFM indentations near the ND for nonlocal deformation mapping. For each indention, a loading force was applied, and once the predetermined set point of the force is reached, a pulse signal was sent out from AFM triggering the start of the AFM modulation and ODMR measurements (the measurement duration is typically 25–30 s). The AFM modulation was implemented by the Ramp mode of the NanoScope software, where a modulation of the tip height was added into the contact (hold) segment of the depth-loading curve.

We used AFM local-depth-loading curves to check the uniformity of the mechanical response of the samples. The observed similar depth-loading curves at various locations on the sample surface around the ND verified the lateral homogeneity of the PDMS films (see Supporting Information Figure S6) and MCF-7 cells (see Supporting Information Figure S15).

### Analysis of the Two-Point ODMR and AFM Data

In each AFM indentation experiment, the ND fluorescence [*P*_on_(*t*) and *P*_off_(*t*) with the switching on- and off-resonance MWs of frequencies  and *f*_off_, see Supporting Information Figure S7 for example] and AFM data [loading *L*(*t*) and indentation depth *d*(*t*)] collections are triggered at the start of the force modulation, where a harmonic rotation of the external magnetic field are applied for calibrations (see Supporting Information Figures S1, S2, and S4). The two-point ODMR signal is obtained as *S* ≡ (*P*_on_ – *P*_off_)/*P*_off_ by normalizing the on-resonance fluorescence with the off resonance one [see Figure 2b for example]. The effect of hydrodynamic drag on the AFM data was corrected by the drag constant in the liquid. Moreover, the effect of cantilever reflection on the fluorescence signal was calibrated by applying an additional oscillation to the laser power. For details of the correction, see Supporting Information Note S1 and Figures S7 and S8.

In order to visualize the oscillation of the AFM and ODMR data by suppressing the shot noise, Figure 2c plots the result by separating the data (measured in 30 s) in continuous segments with a duration of 0.2 s (with integer cycles of each oscillation) and then averaging all the segments. By applying the Fourier transformation, the AFM local data and the two-point ODMR signal were transformed to the frequency domain with frequency *f*^′^ as
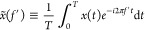
1where  is a complex function and *T* is the total measurement time, as shown in Figure 2d for examples. The amplitude and the phase of each modulation were extracted as the absolute value and the argument of complex number . The amplitude of the ND harmonical rotation  [see Figure 2e for example] was deduced by the ratio of the peaks of the loading to the magnetic modulations [see the bottom panel in Figure 2d] and the known rotation angle amplitude of the magnetic field. The local (nonlocal) phase, i.e., ϕ_AFM_ (ϕ_ND_), was deduced by the difference of the phase between the loading and the depth (ND rotation) modulations.

### Viscoelastic Model upon Point Loading with Surface Tension Effect

With the capillary effect included, an analytical solution was derived for the time-dependent nonlocal deformation *z*(ρ,*t*), upon point loading in half-infinity incompressible linear viscoelastic model.^27^ Under an oscillating point-loading with holding force as *L* = *L*_0_ + δ*L*(*t*), the Fourier transformation of the deformation *z* is written as
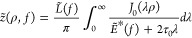
2where *J*_0_(*x*) is the Bessel function,  is the complex modulus of the bulk with the phase , and τ_0_ is the surface tension. The attached ND would be rotating following the deformation on the surface as , where the modulation is written as
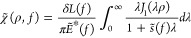
3where  is the frequency-dependent elastocapillary length. After rescaling, the rotation modulation can be written as
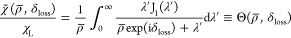
4where  is the normalization factor and  is the rescaled distance. Equation 4 shows that the dynamic nonlocal deformation relies only on the magnitude of the applied force modulation δ*L* and the intrinsic mechanical properties of the material. Supporting Information Figure S9 plots the rescaled amplitude  and the normalized phase lag ϕ_ND_/δ_loss_ = −arg(Θ)/δ_loss_ as functions of the rescaled distance  at different δ_loss_. The two functions are approximately universal functions with only weak dependences on the loss angle δ_loss_. In the capillary limit where  ≪ 1, the normalized amplitude decays as ρ^–1^ and there is no phase lag. In the viscoelasticity limit, where  ≫ 1, the normalized amplitude decays as ρ^–2^ and the phase lag is equal to that of the bulk. In the intermediate case, the decay behavior changes from ρ^–1^ to ρ^–2^, and the nonlocal phase lag increases from 0 to δ_loss_ by increasing ρ from the static capillarity-domaining region () to the dynamic viscoelasticity-domaining region () [see Figure 2g].

### Evaluation of Apparent Complex Modulus

The indentation with a finite-size parabola tip into a homogeneous viscoelastic material is simulated using the Hertz-Sneddon model.^27^ In the oscillatory nanoindentation measurement, the indenter first indents to a settled depth *d*_0_ and is derived by an oscillatory depth δ*d* with the frequency *f*. In the case that δ*d* ≪ *d*_0_,^35^ the apparent complex modulus at such frequency  can be evaluated by the FT data of the oscillatory depth  and loading  as
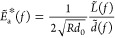
5where *R* is the tip radius with *R* = 25 nm in Figure 3c,d and *R* = 65 nm in Figure 4f,g.

### Oscillatory Rheology Measurement of PDMS

The PDMS bulk sample was prepared by pouring the 60:1 mixture after degassing into a dish with 25 mm diameter to a thickness of ∼3 mm and curing it at 60 °C for 24 h (the same condition as stated above). The bulk mechanics of 60:1 PDMS was measured by oscillatory rheology using a rheometer (MCR 301, Anton-Paar) equipped with a parallel-plate measuring system (PP25 with a diameter of 25 mm). The frequency sweeps were performed in the range 0.1–20 Hz at a constant shear strain amplitude of 1%.

### Extraction of Out-of-Plane Rotation from ODMR Spectra

The time-dependent ODMR spectra were acquired under a static external magnetic field oriented perpendicular to the plane to which the ND was attached. The in-plane rotations (with a rotation axis along the magnetic field) of the ND maintain the angles between the NV centers and the magnetic field, while the out-of-plane rotations (with rotation axes on the *xy*-plane, in the same form of the rotation induced by deformation) rotate the directions of the NV centers relative to the external magnetic field. Therefore, the out-of-plane rotation angle of the ND equals the rotation angle of the magnetic field relative to the NV centers. Such a magnetic field rotation was deduced by fitting the ODMR spectra.

## Supporting Information Available

Experimental setup, sequence for data collection, synchronization between the confocal microscope and the AFM, calibration of the drag constant of the AFM cantilever, approximate universal functions of the viscous-elastocapillary model, analysis and correction of the two-point ODMR and AFM data, and data of deformation measurement and mechanical analysis (PDF)

## References

[ref1] Hurst S.; Vos B. E.; Brandt M.; Betz T. Intracellular Softening and Increased Viscoelastic Fluidity during Division. Nat. Phys. 2021, 17 (11), 1270–1276. 10.1038/s41567-021-01368-z.

[ref2] Darling E. M.; Topel M.; Zauscher S.; Vail T. P.; Guilak F. Viscoelastic Properties of Human Mesenchymally-Derived Stem Cells and Primary Osteoblasts, Chondrocytes, and Adipocytes. J. Biomech. 2008, 41 (2), 454–464. 10.1016/j.jbiomech.2007.06.019. 17825308 PMC2897251

[ref3] Wojcieszyn J. W.; Schlegel R. A.; Wu E. S.; Jacobson K. A. Diffusion of Injected Macromolecules within the Cytoplasm of Living Cells. Proc. Natl. Acad. Sci. U.S.A. 1981, 78 (7), 4407–4410. 10.1073/pnas.78.7.4407. 6945591 PMC319799

[ref4] Kole T. P.; Tseng Y.; Jiang I.; Katz J. L.; Wirtz D. Intracellular Mechanics of Migrating Fibroblasts. Mol. Biol. Cell 2005, 16 (1), 328–338. 10.1091/mbc.e04-06-0485. 15483053 PMC539176

[ref5] Rigato A.; Miyagi A.; Scheuring S.; Rico F. High-Frequency Microrheology Reveals Cytoskeleton Dynamics in Living Cells. Nat. Phys. 2017, 13 (8), 771–775. 10.1038/nphys4104. 28781604 PMC5540170

[ref6] Wang H.; Zhang H.; Da B.; Lu D.; Tamura R.; Goto K.; Watanabe I.; Fujita D.; Hanagata N.; Kano J.; Nakagawa T.; Noguchi M. Mechanomics Biomarker for Cancer Cells Unidentifiable through Morphology and Elastic Modulus. Nano Lett. 2021, 21 (3), 1538–1545. 10.1021/acs.nanolett.1c00003. 33476166

[ref7] Rother J.; Nöding H.; Mey I.; Janshoff A. Atomic Force Microscopy-Based Microrheology Reveals Significant Differences in the Viscoelastic Response between Malign and Benign Cell Lines. Open Biol. 2014, 4 (5), 140046 10.1098/rsob.140046. 24850913 PMC4042852

[ref8] Efremov Y. M.; Okajima T.; Raman A. Measuring Viscoelasticity of Soft Biological Samples Using Atomic Force Microscopy. Soft Matter 2020, 16 (1), 64–81. 10.1039/C9SM01020C. 31720656

[ref9] Krieg M.; Fläschner G.; Alsteens D.; Gaub B. M.; Roos W. H.; Wuite G. J. L.; Gaub H. E.; Gerber C.; Dufrêne Y. F.; Müller D. J. Atomic Force Microscopy-Based Mechanobiology. Nat. Rev. Phys. 2019, 1 (1), 41–57. 10.1038/s42254-018-0001-7.

[ref10] Xia K.; Liu C.-F.; Leong W.-H.; Kwok M.-H.; Yang Z.-Y.; Feng X.; Liu R.-B.; Li Q. Nanometer-Precision Non-Local Deformation Reconstruction Using Nanodiamond Sensing. Nat. Commun. 2019, 10 (1), 3259 10.1038/s41467-019-11252-3. 31332185 PMC6646314

[ref11] Cui Y.; Leong W.-H.; Liu C.-F.; Xia K.; Feng X.; Gergely C.; Liu R.-B.; Li Q. Revealing Capillarity in AFM Indentation of Cells by Nanodiamond-Based Nonlocal Deformation Sensing. Nano Lett. 2022, 22 (10), 3889–3896. 10.1021/acs.nanolett.1c05037. 35507005

[ref12] Style R. W.; Jagota A.; Hui C.-Y.; Dufresne E. R. Elastocapillarity: Surface Tension and the Mechanics of Soft Solids. Annu. Rev. Condens. Matter Phys. 2017, 8 (1), 99–118. 10.1146/annurev-conmatphys-031016-025326.

[ref13] Love A. E. H. A Treatise on the Mathematical Theory of Elasticity; Cambridge University Press, 2013.

[ref14] Jensen K. E.; Style R. W.; Xu Q.; Dufresne E. R. Strain-Dependent Solid Surface Stress and the Stiffness of Soft Contacts. Phys. Rev. X 2017, 7 (4), 041031 10.1103/PhysRevX.7.041031.

[ref15] Style R. W.; Boltyanskiy R.; Che Y.; Wettlaufer J. S.; Wilen L. A.; Dufresne E. R. Universal Deformation of Soft Substrates Near a Contact Line and the Direct Measurement of Solid Surface Stresses. Phys. Rev. Lett. 2013, 110 (6), 066103 10.1103/PhysRevLett.110.066103. 23432280

[ref16] Pham J. T.; Schellenberger F.; Kappl M.; Butt H.-J. From Elasticity to Capillarity in Soft Materials Indentation. Phys. Rev. Mater. 2017, 1 (1), 015602 10.1103/PhysRevMaterials.1.015602.

[ref17] Ding Y.; Wang J.; Xu G.-K.; Wang G.-F. Are Elastic Moduli of Biological Cells Depth Dependent or Not? Another Explanation Using a Contact Mechanics Model with Surface Tension. Soft Matter 2018, 14 (36), 7534–7541. 10.1039/C8SM01216D. 30152838

[ref18] Diz-Muñoz A.; Fletcher D. A.; Weiner O. D. Use the Force: Membrane Tension as an Organizer of Cell Shape and Motility. Trends Cell Biol. 2013, 23 (2), 47–53. 10.1016/j.tcb.2012.09.006. 23122885 PMC3558607

[ref19] Sens P.; Plastino J. Membrane Tension and Cytoskeleton Organization in Cell Motility. J. Phys.: Condens. Matter 2015, 27 (27), 273103 10.1088/0953-8984/27/27/273103. 26061624

[ref20] Feng X.; Leong W.-H.; Xia K.; Liu C.-F.; Liu G.-Q.; Rendler T.; Wrachtrup J.; Liu R.-B.; Li Q. Association of Nanodiamond Rotation Dynamics with Cell Activities by Translation-Rotation Tracking. Nano Lett. 2021, 21 (8), 3393–3400. 10.1021/acs.nanolett.0c04864. 33847115

[ref21] Guo M.; Ehrlicher A. J.; Jensen M. H.; Renz M.; Moore J. R.; Goldman R. D.; Lippincott-Schwartz J.; Mackintosh F. C.; Weitz D. A. Probing the Stochastic, Motor-Driven Properties of the Cytoplasm Using Force Spectrum Microscopy. Cell 2014, 158 (4), 822–832. 10.1016/j.cell.2014.06.051. 25126787 PMC4183065

[ref22] Miller B. S.; Bezinge L.; Gliddon H. D.; Huang D.; Dold G.; Gray E. R.; Heaney J.; Dobson P. J.; Nastouli E.; Morton J. J. L.; McKendry R. A. Spin-Enhanced Nanodiamond Biosensing for Ultrasensitive Diagnostics. Nature 2020, 587 (7835), 588–593. 10.1038/s41586-020-2917-1. 33239800

[ref23] McGuinness L. P.; Yan Y.; Stacey A.; Simpson D. A.; Hall L. T.; Maclaurin D.; Prawer S.; Mulvaney P.; Wrachtrup J.; Caruso F.; Scholten R. E.; Hollenberg L. C. L. Quantum Measurement and Orientation Tracking of Fluorescent Nanodiamonds inside Living Cells. Nat. Nanotechnol. 2011, 6 (6), 358–363. 10.1038/nnano.2011.64. 21552253

[ref24] Igarashi R.; Sugi T.; Sotoma S.; Genjo T.; Kumiya Y.; Walinda E.; Ueno H.; Ikeda K.; Sumiya H.; Tochio H.; Yoshinari Y.; Harada Y.; Shirakawa M. Tracking the 3D Rotational Dynamics in Nanoscopic Biological Systems. J. Am. Chem. Soc. 2020, 142 (16), 7542–7554. 10.1021/jacs.0c01191. 32285668

[ref25] Hui C.-Y.; Jagota A. Effect of Surface Tension on the Relaxation of a Viscoelastic Half-Space Perturbed by a Point Load. J. Polym. Sci., Part B: Polym. Phys. 2016, 54 (2), 274–280. 10.1002/polb.23920.

[ref26] Karpitschka S.; Das S.; van Gorcum M.; Perrin H.; Andreotti B.; Snoeijer J. H. Droplets Move over Viscoelastic Substrates by Surfing a Ridge. Nat. Commun. 2015, 6 (1), 7891 10.1038/ncomms8891. 26238436 PMC4532859

[ref27] Sneddon I. N. The Relation between Load and Penetration in the Axisymmetric Boussinesq Problem for a Punch of Arbitrary Profile. Int. J. Eng. Sci. 1965, 3 (1), 47–57. 10.1016/0020-7225(65)90019-4.

[ref28] Mahaffy R. E.; Shih C. K.; MacKintosh F. C.; Käs J. Scanning Probe-Based Frequency-Dependent Microrheology of Polymer Gels and Biological Cells. Phys. Rev. Lett. 2000, 85 (4), 880–883. 10.1103/PhysRevLett.85.880. 10991422

[ref29] Wu P.-H.; Aroush D. R.-B.; Asnacios A.; Chen W.-C.; Dokukin M. E.; Doss B. L.; Durand-Smet P.; Ekpenyong A.; Guck J.; Guz N. V.; Janmey P. A.; Lee J. S. H.; Moore N. M.; Ott A.; Poh Y.-C.; Ros R.; Sander M.; Sokolov I.; Staunton J. R.; Wang N.; Whyte G.; Wirtz D. Comparative Study of Cell Mechanics Methods. Nat. Methods 2018, 15 (7), 491–498. 10.1038/s41592-018-0015-1. 29915189 PMC6582221

[ref30] Calzado-Martín A.; Encinar M.; Tamayo J.; Calleja M.; San Paulo A. Effect of Actin Organization on the Stiffness of Living Breast Cancer Cells Revealed by Peak-Force Modulation Atomic Force Microscopy. ACS Nano 2016, 10 (3), 3365–3374. 10.1021/acsnano.5b07162. 26901115

[ref31] Ren K.; Gao J.; Han D. AFM Force Relaxation Curve Reveals That the Decrease of Membrane Tension Is the Essential Reason for the Softening of Cancer Cells. Front. Cell Dev. Biol. 2021, 9, 663021 10.3389/fcell.2021.663021. 34055793 PMC8152666

[ref32] Morris C. E.; Homann U. Cell Surface Area Regulation and Membrane Tension. J. Membr. Biol. 2001, 179 (2), 79–102. 10.1007/s002320010040. 11220366

[ref33] Kollmannsberger P.; Fabry B. Linear and Nonlinear Rheology of Living Cells. Annu. Rev. Mater. Res. 2011, 41 (1), 75–97. 10.1146/annurev-matsci-062910-100351.

[ref34] Sollich P.; Lequeux F.; Hébraud P.; Cates M. E. Rheology of Soft Glassy Materials. Phys. Rev. Lett. 1997, 78 (10), 2020–2023. 10.1103/PhysRevLett.78.2020.

[ref35] Ting T. C. T. Contact Problems in the Linear Theory of Viscoelasticity. J. Appl. Mech. 1968, 35 (2), 248–254. 10.1115/1.3601188.

