# Supplementary material for: Nanodiamond-Based Spatial–Temporal Deformation Sensing for Cell Mechanics
Source: ACS Nano. 2025 Apr 2;19(14):13740–51. doi: 10.1021/acsnano.4c15003 (PMC12004926; doi:10.1021/acsnano.4c15003)
Supplement: Supplementary file 1 — nn4c15003_si_001.pdf [file nn4c15003_si_001.pdf]

Supporting Information

for

Nanodiamond-based spatial-temporal deformation sensing for cell mechanics

Yue Cui[2,1+], Weng-Hang Leong[3,1+], Guoli Zhu[1+], Ren-Bao Liu[1,4,5,6], Quan Li[1,4,5*]*

[+]*These authors contributed equally: Yue Cui, Weng-Hang Leong, Guoli Zhu*

1. Department of Physics, The Chinese University of Hong Kong, Shatin, New Territories, Hong Kong 999077, China

2. Quantum Science Center of Guangdong-Hong Kong-Macao Greater Bay Area (Guangdong), Shenzhen 518045, China

3. Department of Engineering Science, Faculty of Innovation Engineering, Macau University of Science and Technology, Taipa, Macao 999078, China

4. Centre for Quantum Coherence, The Chinese University of Hong Kong, Shatin, New Territories, Hong Kong 999077, China

5. State Key Laboratory of Quantum Information Technologies and Materials, The Chinese University of Hong Kong, Shatin, New Territories, Hong Kong 999077, China

6. New Cornerstone Science Laboratory, The Chinese University of Hong Kong, Shatin, New Territories, Hong Kong 999077, China

**Corresponding authors. E-mails: rbliu@cuhk.edu.hk and liquan@cuhk.edu.hk*

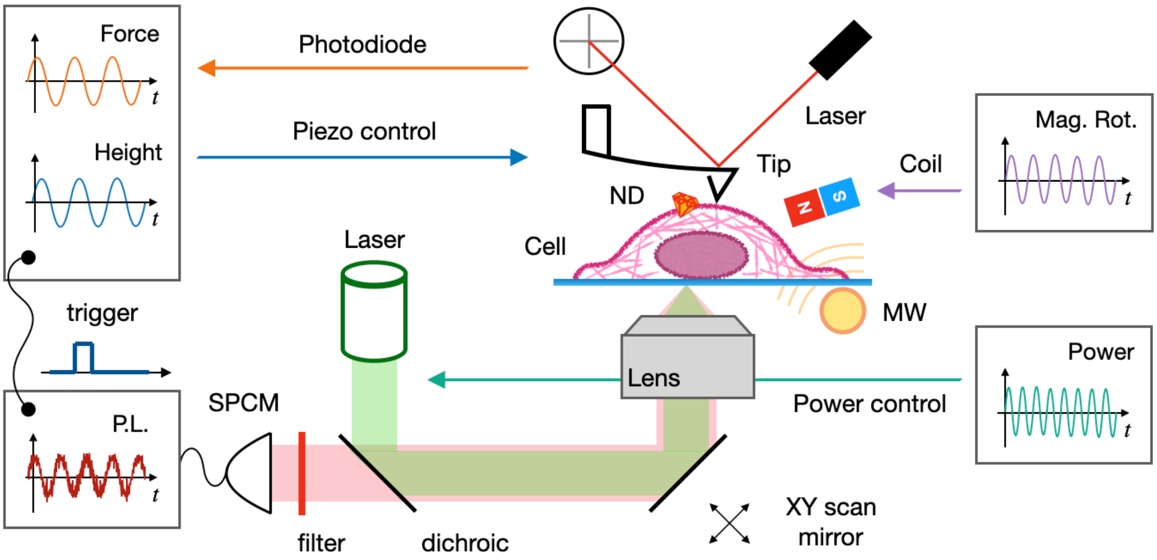

Supplementary Figure S1 | Schematic of the confocal-atomic force microscope (AFM) setup. *Confocal microscope:* a 532 nm laser was adopted (MGL-III-532-200 mW, CNI). A Nikon 100x (1.45 NA) oil immersion objective lens was used to collect the fluorescence of nanodiamonds (NDs), which was then detected by a single-photon counting module (SPCM, SPCM-AQRH-15-FC, Excelitas). *AFM*: the AFM scanning head (BioScope Resolve, Bruker) was mounted on the confocal microscope to measure the topography and to apply indentation. High Speed Data Capture (HSDC) function (Nanoscope, Bruker) was used for AFM data acquisition. *Optically detected magnetic resonance (ODMR) spectrum measurement:* Microwave (MW) sources (N5171B EXG Signal Generator, Keysight & WB-SG1-8G, Taobao) and an amplifier (ZHL-16W-43-S+, Mini-Circuit) were used to generate microwave (see Supplementary Figure S5 for details). A coil was put on the objective lens to rotate the electromagnetic field applied to the sample (see Supplementary Figure S4 for details). NIDAQ (PCIe-6363, National Instrument) counted the single-photon signal from the SPCM, and controlled the laser and the coil to modulate the laser power and the direction of the external magnetic field during indentation. Force modulation of the AFM tip was started by the NanoScope V Controller of the AFM microscope while a pulse signal (a negative-going pulse with width of 20 μs) was sent out and triggered the fluorescence and AFM data collections.

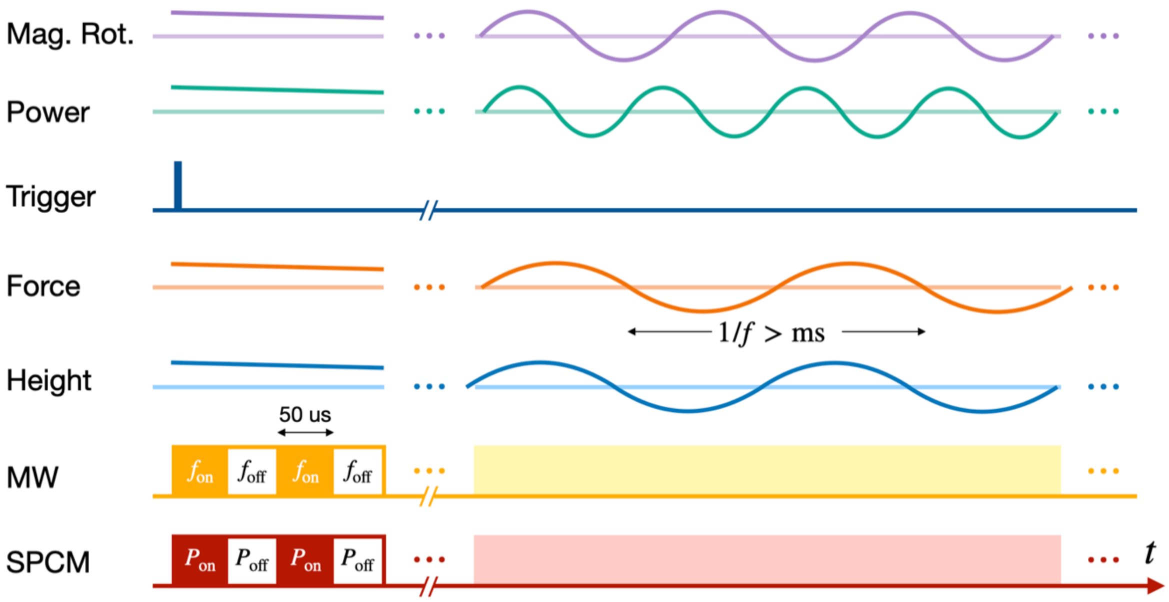

Supplementary Figure S2 | Sequence for data collection in the dynamic nonlocal response measurement. The modulation of the external magnetic field direction and laser power was consistently applied during the oscillating AFM indentation to calibrate the rotation angle magnitude of the NDs and correct for laser fluctuations (for more information, refer to Supplementary Note 1). The collection of AFM data, including force and corresponding tip heights measurements, was triggered by a pulse signal sent by the AFM, coinciding with the start of height modulation of the AFM tip. Simultaneously, the recording of ODMR signals was triggered by the same pulse signal, which alternated between on- and off-resonance microwave frequencies, $\{f_i\}_{on}$ and f_{off} (see Supplementary Figure S5), and the corresponding fluorescence counting, P_{on} and P_{off}, respectively.

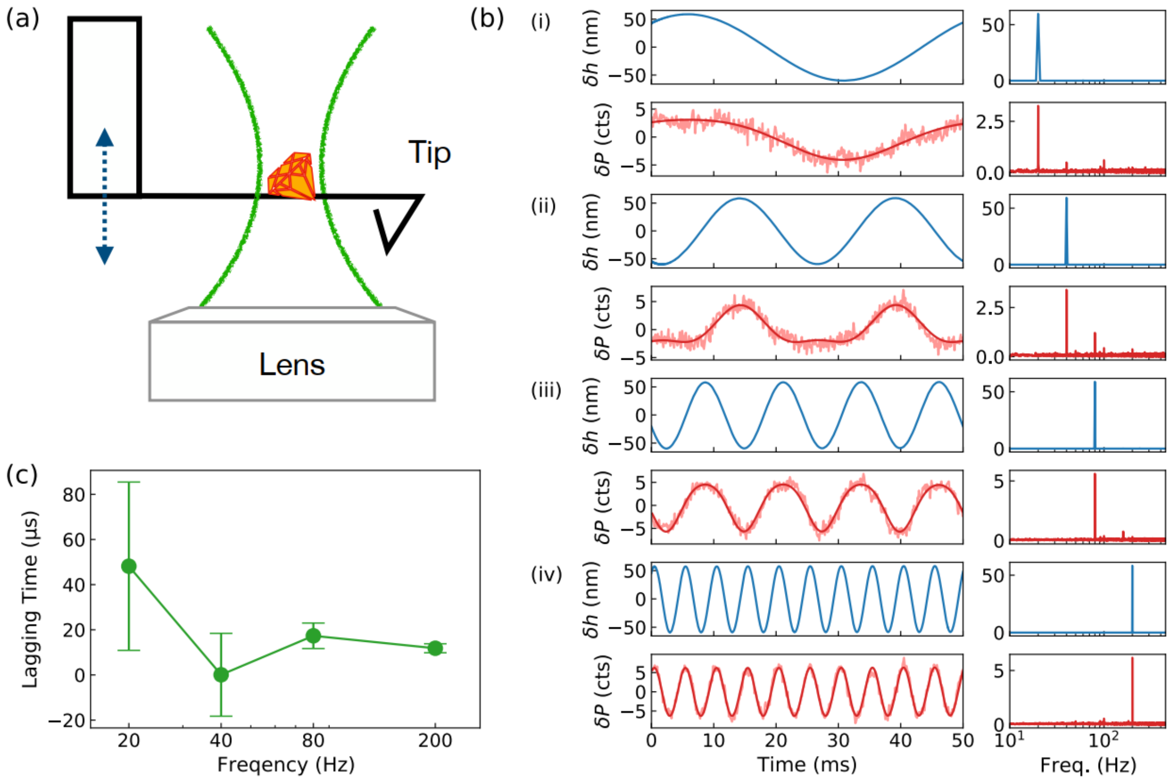

Supplementary Figure S3 | Synchronization between the confocal microscope and the AFM. (a) Scheme illustrating the preliminary experiment conducted to verify the synchronization between the confocal and AFM systems. NDs were drop-casted and positioned on the cantilever. Non-contact height modulations of the AFM tip were performed above the substrate, causing fluorescence oscillations of the ND that were captured by the confocal setup. (b) i to iv: The oscillating height data (h, captured by the AFM) and the ND fluorescence data (P, captured by the confocal setup) at different height modulation frequencies f of 20, 40, 80, and 200 Hz, respectively. The Fourier transform results of the data are displayed in the right panel of (b). (c) The lagging time between the AFM (height data) and the confocal (fluorescence data) represented as a function of modulation frequency. The lagging time was calculated by multiplying the phase difference (obtained from the Fourier results) by the periodicity ($1/f$).

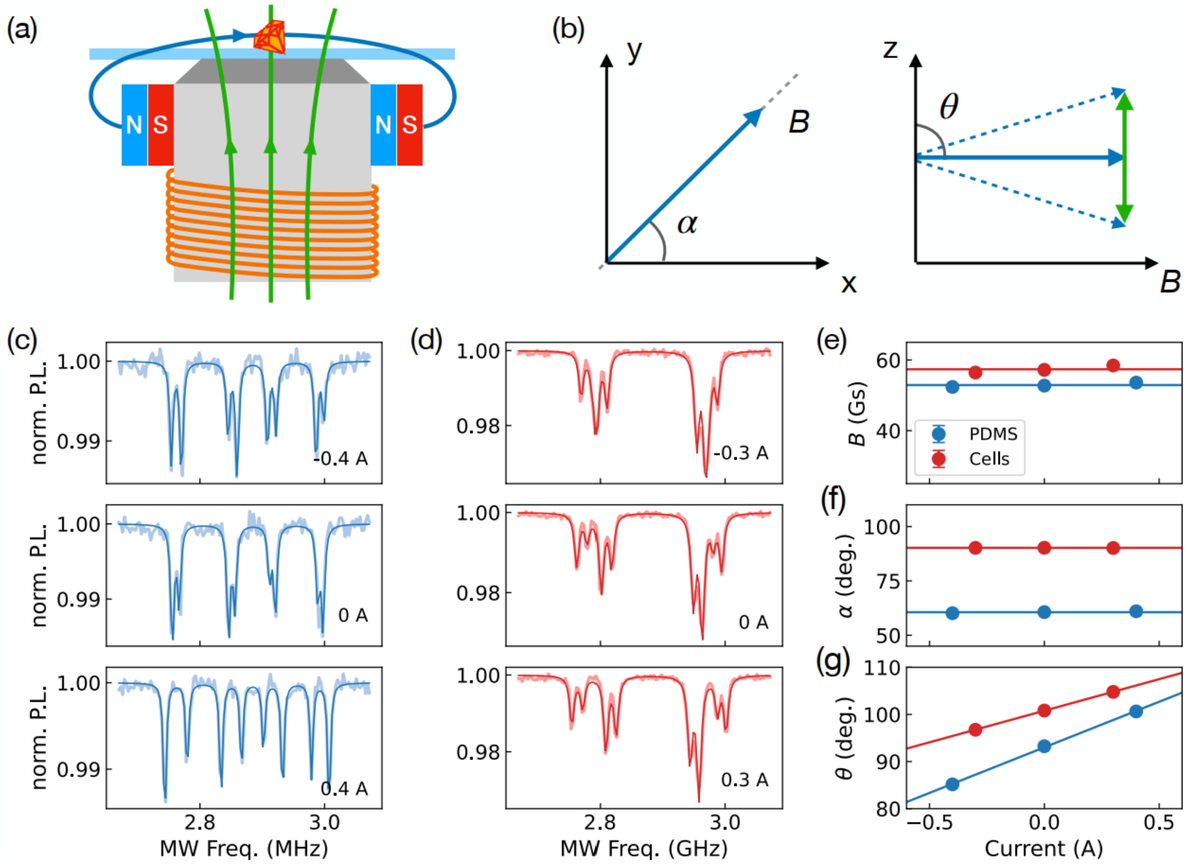

Supplementary Figure S4 | Control and calibration of external magnetic field. (a) The external magnetic field generated by a pair of permanent magnets and a magnetic coil fixed on the objective lens. The current through the coil is adjustable. (b) The static external magnetic field from the permanent magnets (blue arrows) and the adjustable field from the coil (the green arrows showing its range) add up to a total magnetic field that can be rotated in the range indicated by the blue dashed arrows. (c) and (d) The ODMR spectra obtained in the calibration of the external magnetic fields with a bulk diamond for the PDMS and the live cells experiments with different currents through the coil. The fitting of the ODMR spectrum under an external magnetic field is also plotted. (e) to (g) The magnitude B, azimuthal angle α and the polar angle θ as functions of the current though the coil. The magnitude and the azimuthal angle were approximately unchanged (with means of $\bar{B}$ =52.9 Gs and $\bar{\alpha} = 60.6\ deg.$ for the PDMS cases; and $\bar{B}$ =57.4 Gs and $\bar{\alpha} = 90.2\ deg.$ for the live cell cases), while the polar angle θ varied by the current with the slope of 19.4 $deg./A$ and 13.4 $deg./A$ in the PDMS and the live cell experiments, respectively.

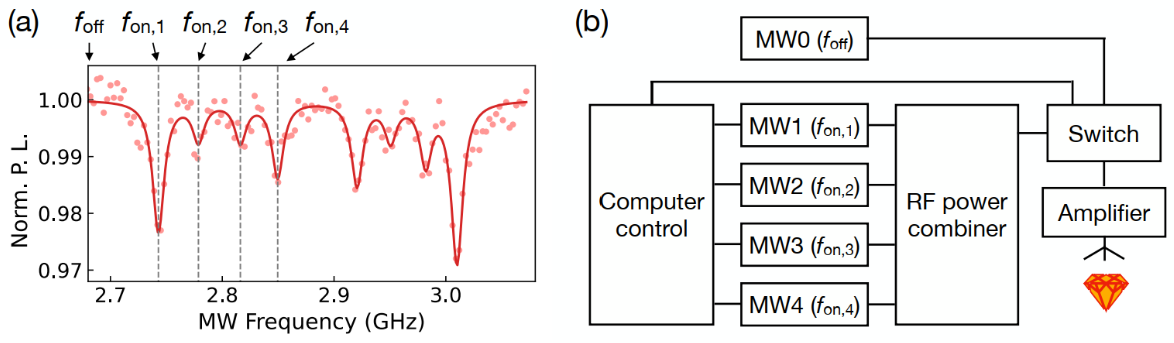

Supplementary Figure S5 | Scheme of the four MW generators setup. (a) A typical ODMR spectrum of the ND located on the PDMS as illustrated in Fig. 2(a) in the main text. The red line is the fitting results. The off-resonance frequency f_{off} was set to 2.68 GHz. The dashed lines indicate the four on-resonance frequencies $f_{on,i}, i = 1\ to\ 4$, as estimated from the fitting results. (b) Scheme of the MW generators. The four MWs of on-resonance frequencies were mixed by a power combiner. A switch was used to switch the MW between the on- and off-resonance ones, and was controlled by the NIDAQ and the computer.

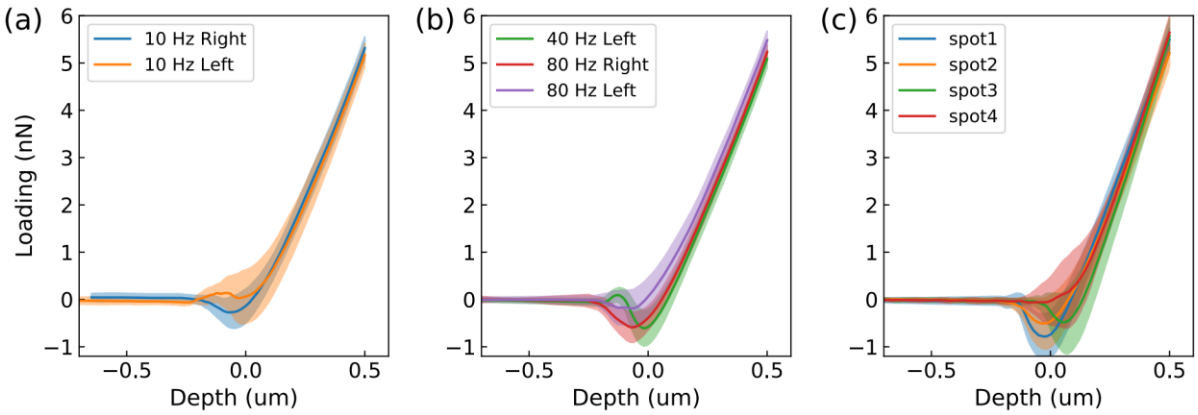

Supplementary Figure S6 | Approach depth-loading curves of the AFM indentations on PDMS. Averaged depth-loading curves of the local deformations under a constant indentation rate ($600\ nm\ s^{-1}$) of (a) the 200 indentations as indicated in Fig. 2(a) in the main text, (b) the 250 indentations as shown in Supplementary Figures S10, and (c) the 320 indentations in Supplementary Figure S11. The error bars (given by the shadow) show the standard deviations of the measurements.

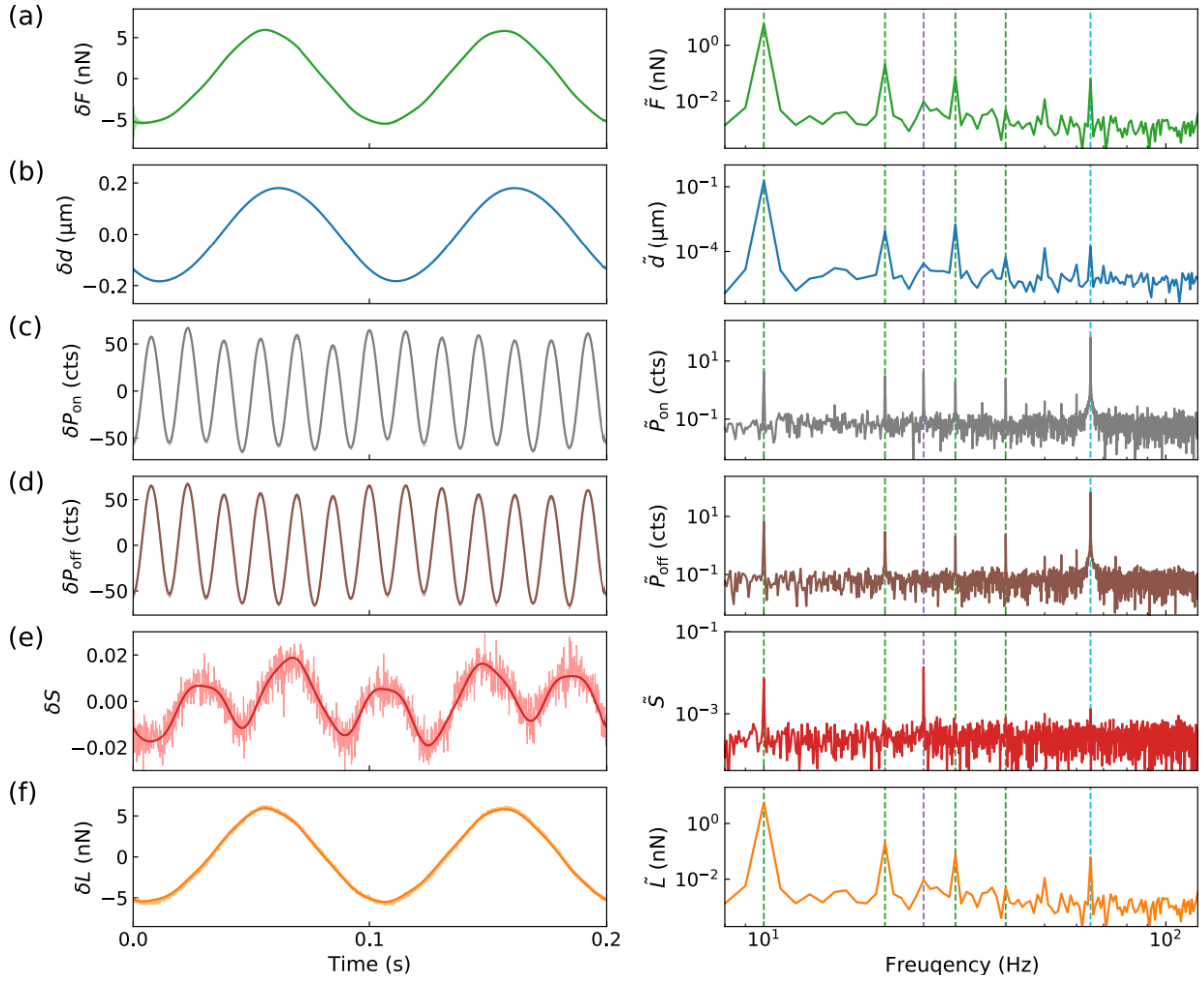

Supplementary Figure S7 | Typical AFM and ODMR data of the dynamic nonlocal mechanical response measurement. Left: The time averaging and Right: the Fourier transform (FT) of (a) the force F and (b) the depth d of the AFM tip, (c) the on resonant fluorescence P_{on} and (d) the off resonant fluorescence P_{off}, (e) the two point ODMR signal S and (f) the loading L after the correction of hydrodynamic drag (for details, see Supplementary Note 1) at the indentation location indicated by the black arrow in Fig. 2(a) in the main text. The colored dashed lines in the right panel indicate the harmonical peaks of the FT signal induced by the oscillations of AFM tip (green), magnetic field (purple) and laser power (cyan).

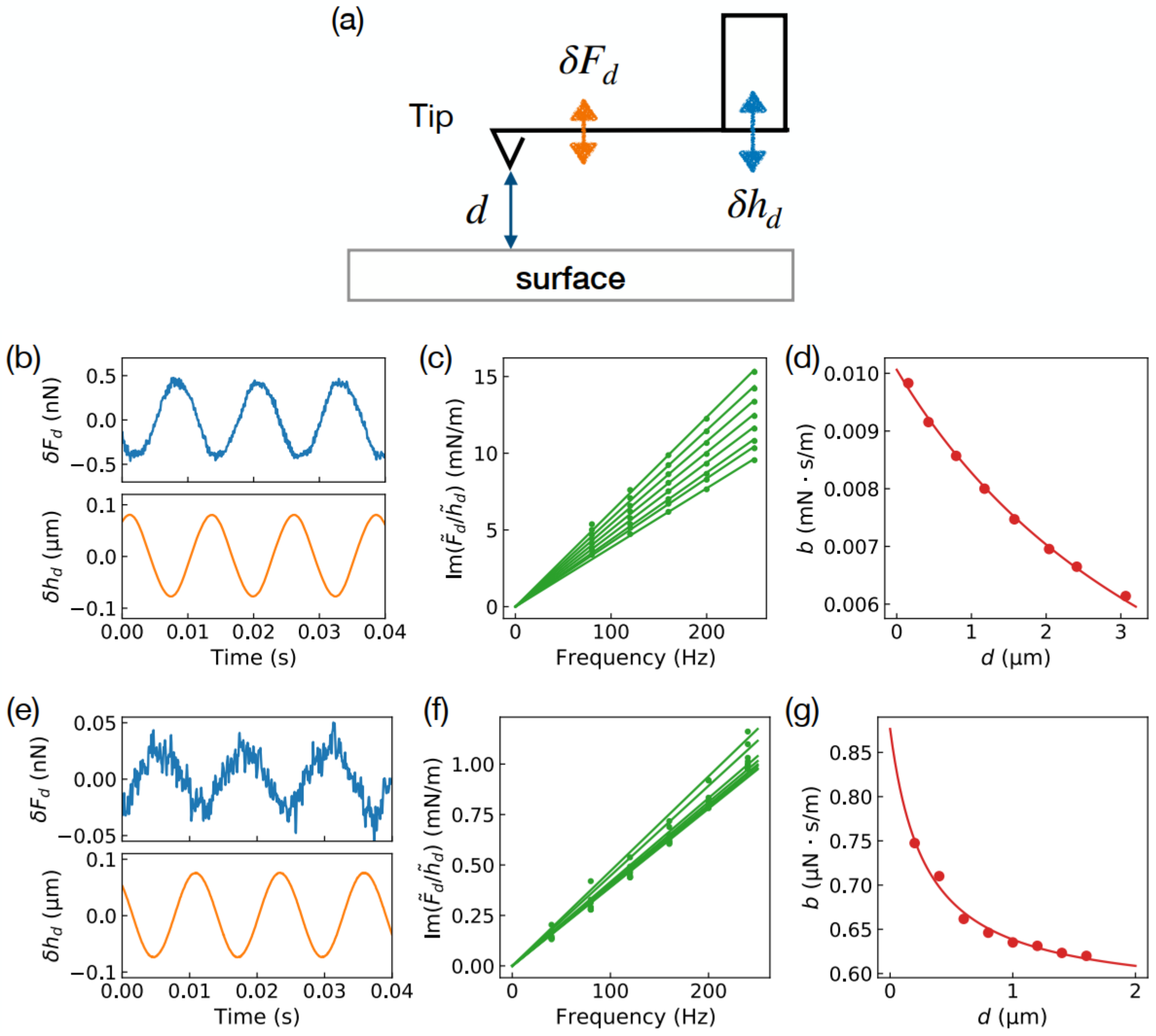

Supplementary Figure S8 | Calibration of the drag constant of the AFM cantilevers. (a) Scheme of the preliminary experiment for the drag coefficient calibration, see Supplementary Note 1 for details. (b) A typical drag force $F_d(t)$ and height modulation $h_d(t)$ with frequency of 80 Hz of the DNP-10-A tip (in the PDMS experiments). (c) The imaginary part of the ratio between the peak value of the Fourier transform of the drag force and the height as functions of the modulation frequency with different separation d between the tip and the surface. The green lines are the linear fitting of the data. (d) the drag coefficient [slopes in (c)] as a function of the separation d. The red line is the fitting results. (e) to (g) the similar plots as those in (b) to (d) in the live cell experiments. The drag coefficients for the DNP-10-A tip (in the PDMS experiments) and PFQNM-LC-A-CAL tip (in the cell experiments) were calibrated to be 1.0×10^{-5} and $8.7 \times 10^{-7}\ N\ s\ m^{-1}$ at $d = 0$, respectively.

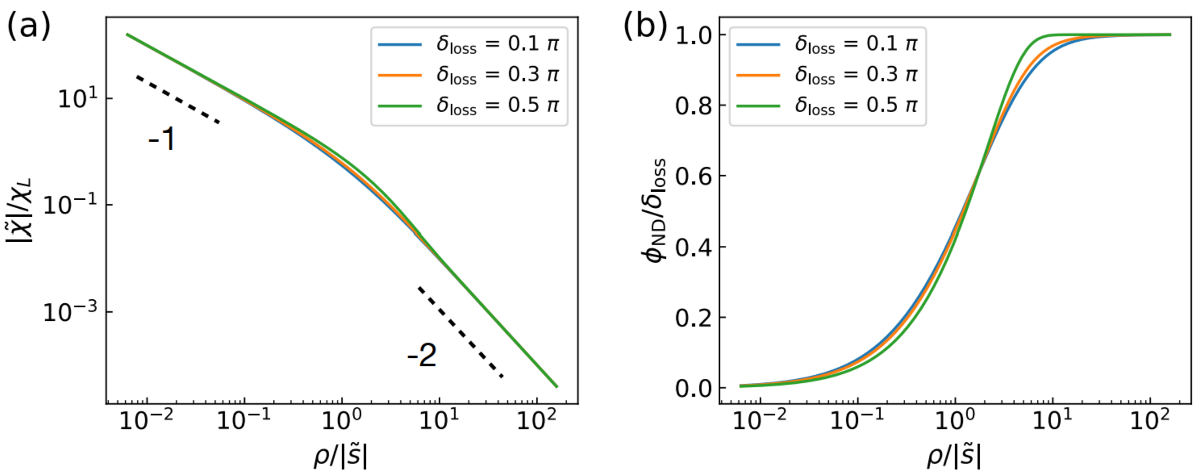

Supplementary Figure S9 | The approximately universal functions of the viscous-elastocapillary model. (a) The modulus $|\Theta| = |\tilde{\chi}|/\chi_L$ and (b) the normalized phase lag $-arg(\Theta)/\delta_{loss}$ of the approximately universal functions (see Methods in the main text) of the rescaled distance $\bar{\rho} = \rho/|\tilde{s}|$ at different δ_{loss}. The dashed lines in a indicate the asymptotic behavior of $|\Theta| \approx \bar{\rho}^{-1}$ and $|\Theta| \approx \bar{\rho}^{-2}$ in the capillary ($\bar{\rho} \ll 1$, where the surface tension effect dominates) and the viscoelasticity ($\bar{\rho} \gg 1$, where the bulk effect dominates) limits.

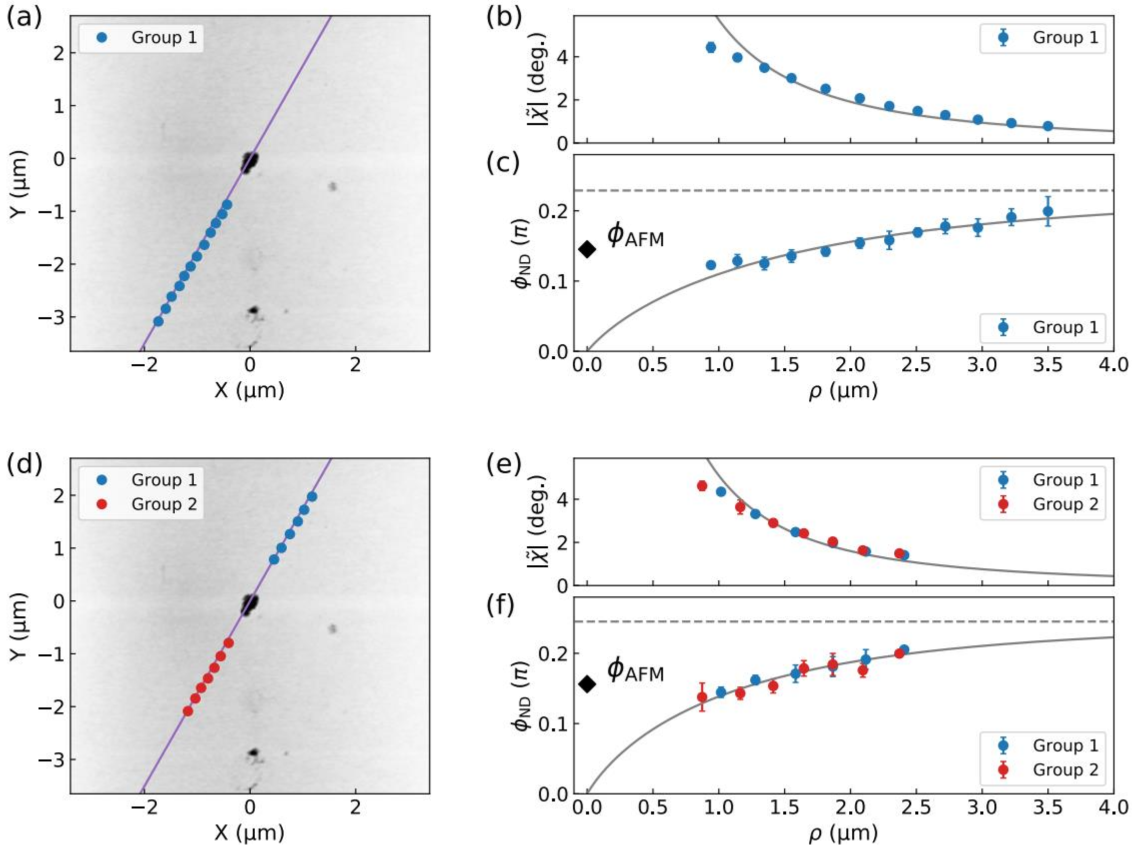

Supplementary Figure S10 | The dynamic nonlocal response of PDMS under indentation at frequencies 40 & 80 Hz. (a) AFM image as shown in Fig. 2(a) in the main text. The dots represent the indentation locations of the AFM tip around the ND. The purple line indicates the direction of the external magnetic field $\boldsymbol{B}$. (b) The amplitude of the oscillatory rotation angle ($|\tilde{\chi}|$) and (c) The nonlocal phase lag (ϕ_{ND}) as functions of the distance ρ between the indentation spot and the ND with modulation frequency of 40 Hz. The simulation results of the linear viscoelastic model including the surface tension effect are plotted by the grey lines. The local phase lag ϕ_{AFM} deduces from the AFM data is drawn by the rhombus at zero distance, while the bulk loss angle δ_{loss} is indicated by the grey dashed line. (d) to (f) the similar plots as those in (a) to (c) with modulation frequency of 80 Hz. The error bars in (b), (c), (e) and (f) are the standard derivations of the repeated measurements.

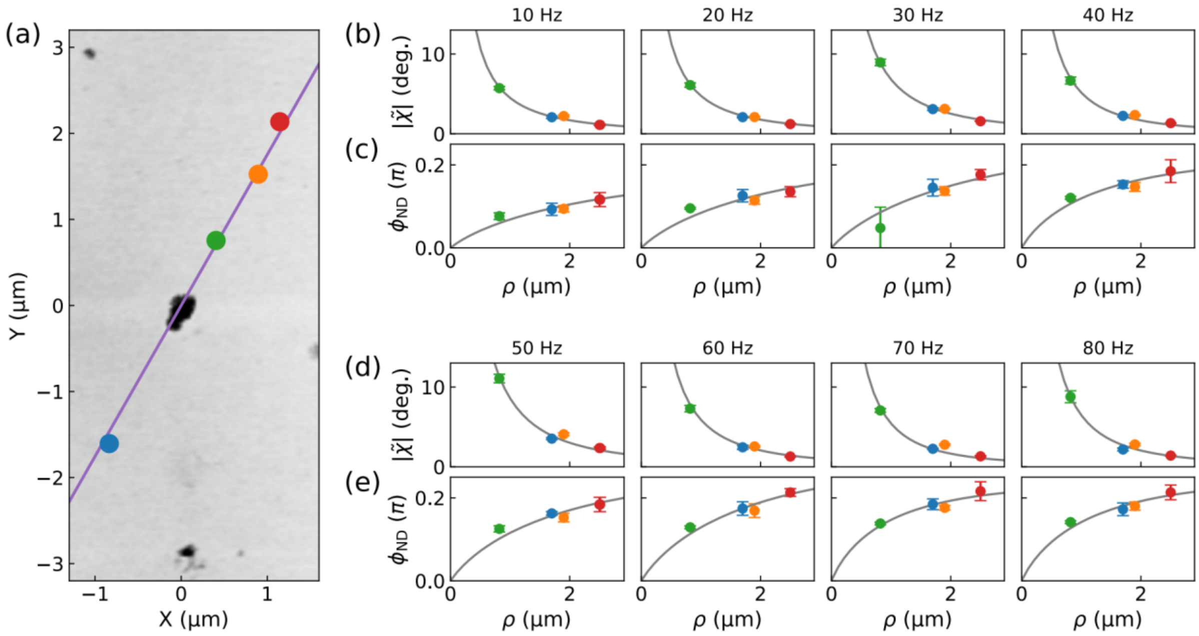

Supplementary Figure S11 | The dynamic nonlocal response of PDMS with different indentation frequencies ranging from 10 to 80 Hz. (a) The same AFM image of the PDMS surface as shown in Fig. 2(a) in the main text. The dots represent the indentation locations of the AFM tip around the ND. The purple line indicates the direction of the external magnetic field $\boldsymbol{B}$. (b) & (d) The amplitude of the oscillatory rotation angle ($|\tilde{\chi}|$), and (c) & (e), The nonlocal phase lag (ϕ_{ND}) as functions of the distance ρ between the indentation spot and the ND with different modulation frequencies ranging from 10 to 80 Hz. The simulation results of the linear viscoelastic model including the surface tension effect are plotted by the grey lines. The error bars in (b) to (e) are the standard derivation of the repeated measurements.

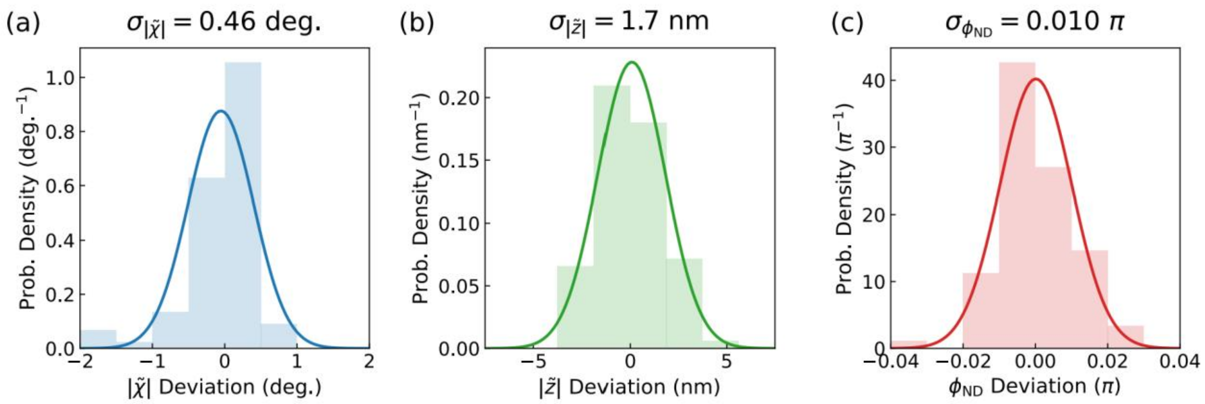

Supplementary Figure S12 | Comparison between the experimental and simulation results of PDMS measurement. The histogram of the deviations between the experimental and theoretical results of (a) the rotation oscillation amplitude $|\tilde{\chi}|$, (b) the non-local deformation amplitude $|\tilde{z}|$ and (c) the nonlocal phase lag ϕ_{ND} for the PDMS measured in Figs. 3(a) and 3(b) of the main text. The experimental results $\tilde{\chi}$ and $\tilde{z}$ were obtained by ODMR measurements and deformation reconstruction via integration (see Ref. [10] in the main text). The theoretical results (such as the grey lines in Figs. 3(a) and 3(b) in the main text) were calculated by using the viscoelastic model (see Methods in the main text). The solid lines are the fitting results with Gaussian distribution, with standard deviations shown at the top of each figure.

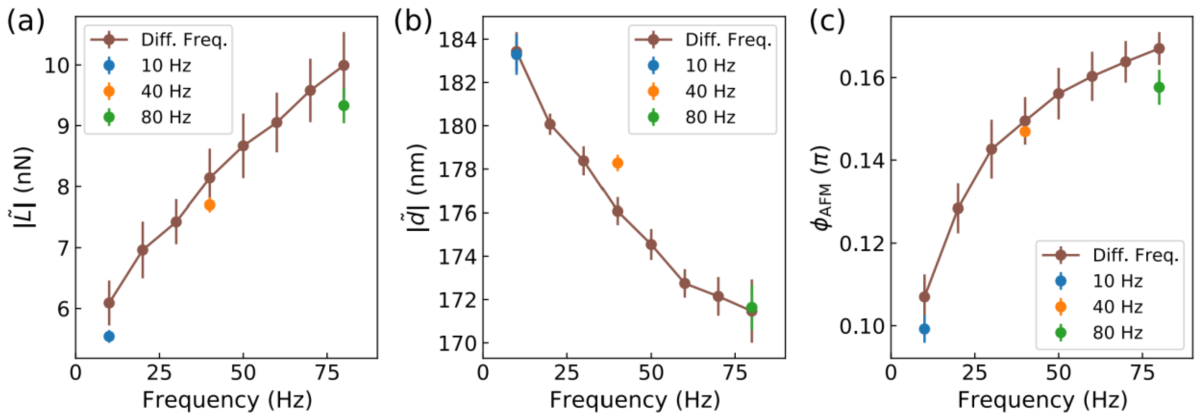

Supplementary Figure S13 | The local indentation data of the PDMS. The magnitude of the modulation of (a) the loading and (b) the indentation depth as functions of the modulation frequencies for the indentations shown in Fig. 2(a) in the main text (10 Hz), Supplementary Figure S10 (40 and 80 Hz), and Supplementary Figure S11 (10-80 Hz). (c) The corresponding phase lag ϕ_{AFM} between the loading and the depth of the indentations. The error bars are the standard derivations of the repeated measurements.

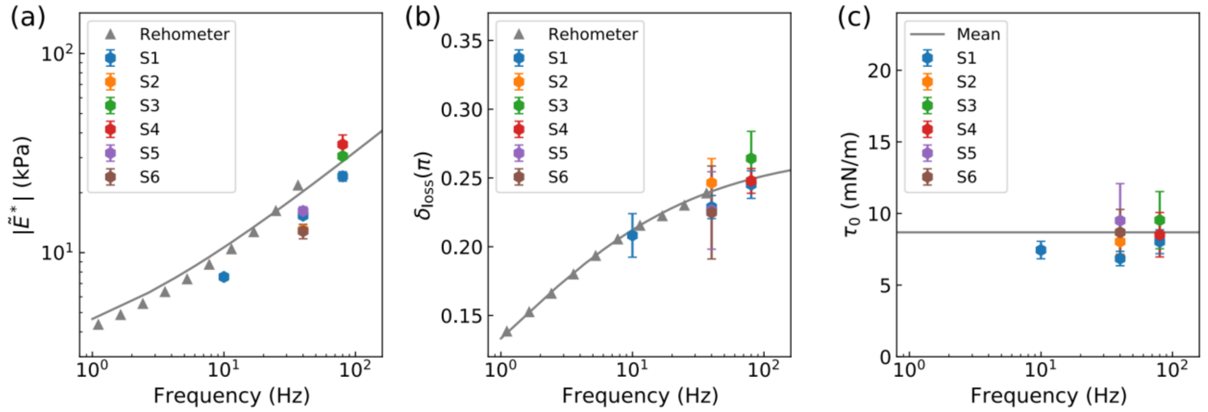

Supplementary Figure S14 | The mechanical properties of different PDMS samples. (a) The evaluated magnitude $|\tilde{E}^*|$ and (b) the loss angle δ_{loss} of the complex modulus as functions of the frequency f for different PDMS samples. The blue dots (S1) are the complex modulus as shown in Fig. 3 in the main text. The grey triangles and lines plot the complex modulus of PDMS measured by rheometer and the corresponding fitting results evaluated by using the power-law function as shown in Fig. 3 in the main text. (c) The surface tension τ_0 deduced in the different experiments. The line indicates the mean value. The error bars are fitting errors.

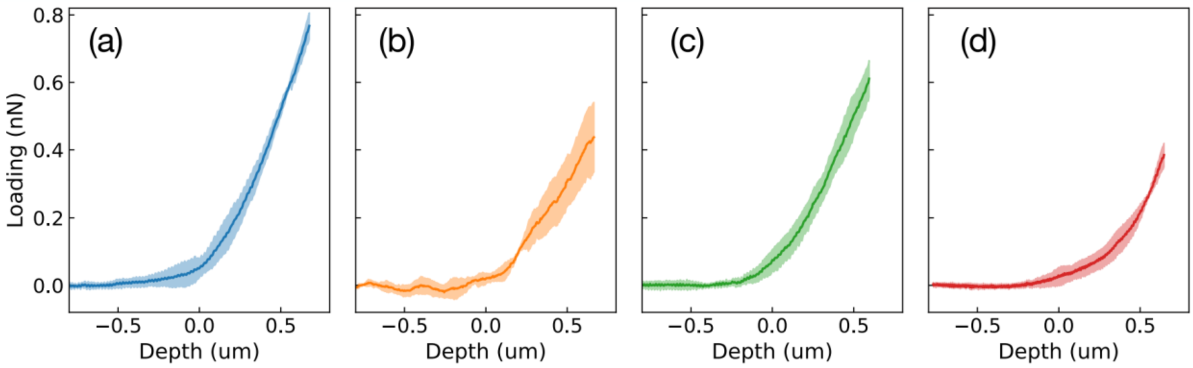

Supplementary Figure S15 | Approach depth-loading curves of the indentation on the live cells. (a) to (d) Averaged depth-loading curves of the local deformations under a constant indentation rate (600 $nm\ s^{-1}$) on the four live cells as illustrated in Fig. 4 in the main text. The error bars (given by the shadow) show the standard deviations of the measurements.

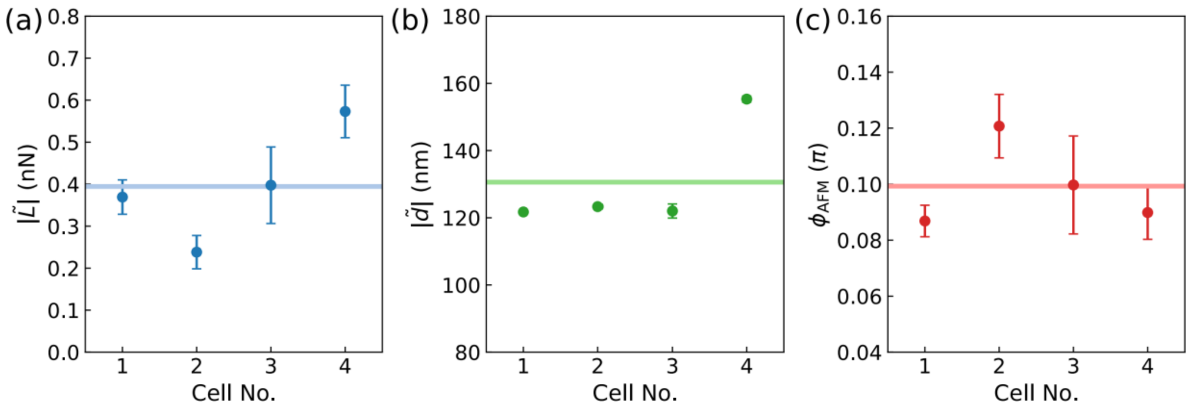

Supplementary Figure S16 | The local indentation data on cells. The amplitudes of the modulation of (a) the loading L and (b) the indentation depth d for the 40 Hz modulation on the four cells in Fig. 4 in the main text. (c) The corresponding phase lag ϕ_{AFM} between the loading and the depth of the indentations. The error bars are the standard derivation of the repeated measurements.

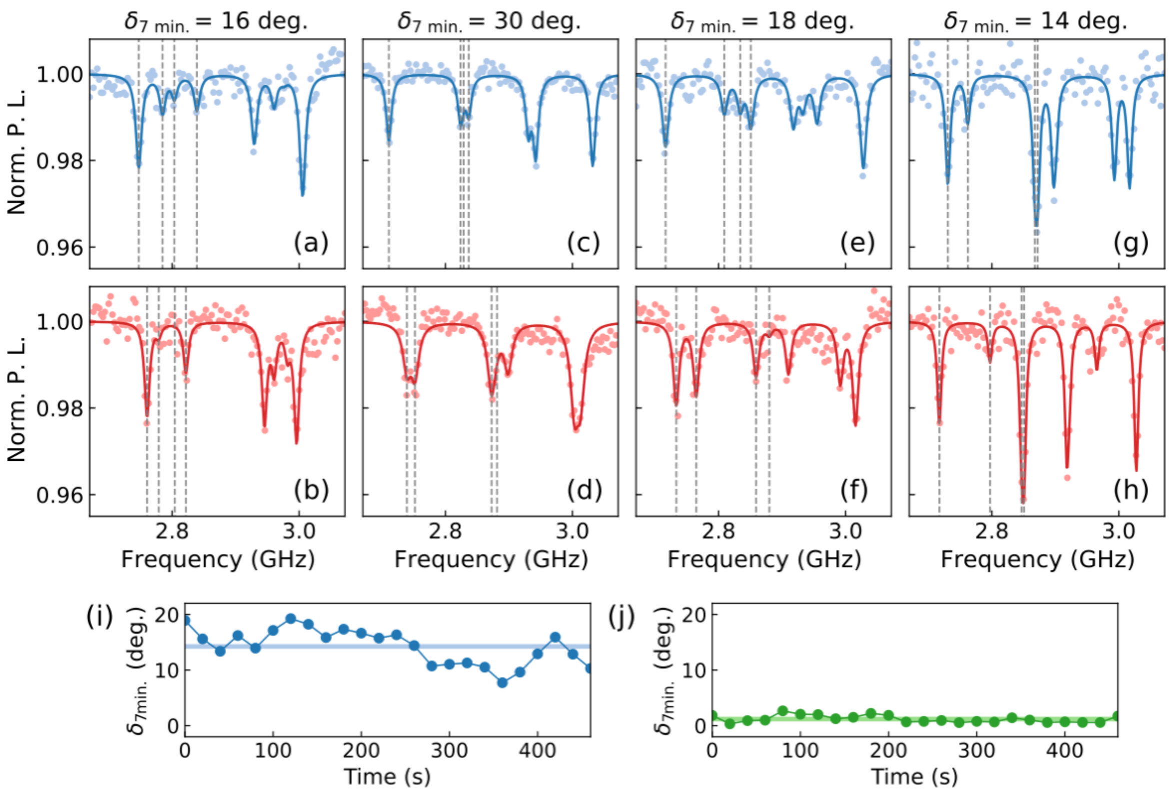

Supplementary Figure S17 | The change in ODMR spectra of the ND attached on the live cells in a duration of 7 minutes. (a) and (b) The ODMR spectra of the attached ND on the live cell 1 in Fig. 4 in the main text obtained after all the indentations. The time interval between the acquisitions of the two ODMR spectra was 7 minutes. The colored lines are the fitting results. The grey dashed lines indicate the on-resonance frequencies evaluated by fitting. The minimum rotation angle $\delta_{7\ min.}$ of the ND within the 7 min is estimated by the angle between the static external magnetic fields measured by the rotating ND [deduced by the fitting, similar to those in Supplementary Figures S4(c) and (d)] before and after the time interval. (c)&(d), (e)&(f) and (g)&(h) are similar to (a)&(b), but for the other three live cell experiments labelled as Cell 2-4. (i) The typical minimum rotation angle $\delta_{7\ min.}$ of the ND within 7 min time duration on live MCF-7 cells obtained from the time-dependent ODMR spectrum in Fig. 4(c) of the main text. The blue line is the mean value. (j) is similar to (i) but is the result measured after the cell died.

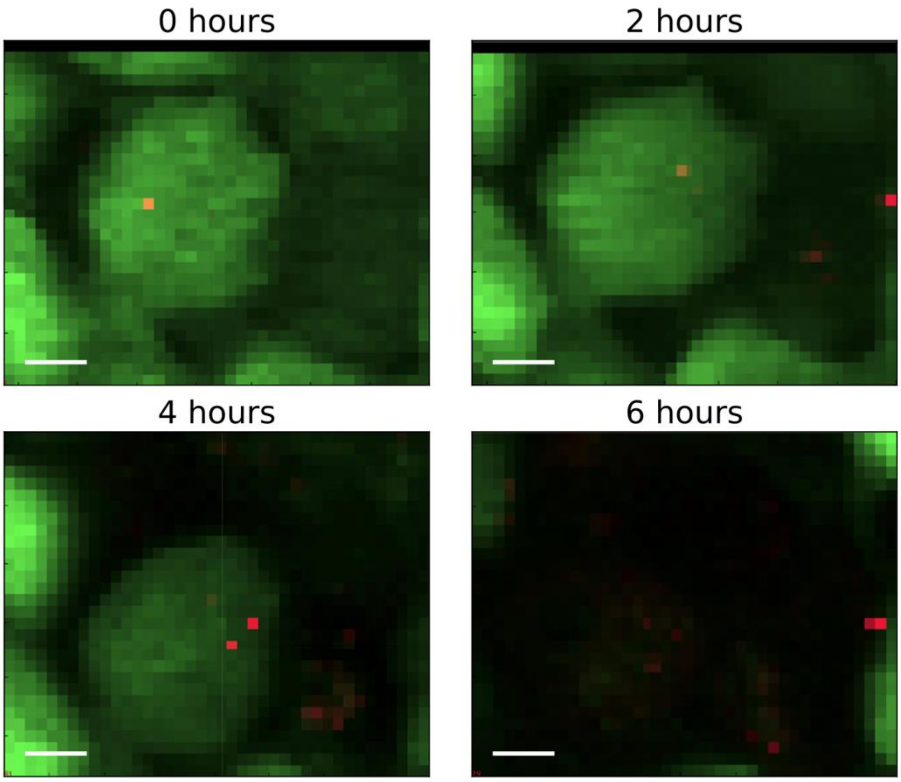

Supplementary Figure S18 | The Change in cell viability during typical ODMR measurement of nanodiamonds on a live MCF-7 cell with laser and microwave applied. The fluorescence signal from the viability assay BCECF AM (Thermo Fisher) works as an indicator of cell viability.

Supplementary Note 1 | Analysis and correction of the two-point ODMR and AFM data

In each AFM indentation experiment, the collection of the ND fluorescence data ($P_{\mathrm{on}}(t)$ and $P_{\mathrm{off}}(t)$ for MW frequencies being on-resonance $\{f_i\}_{\mathrm{on}}$ and off-resonance f_{off}, respectively) and AFM data (force $F(t)$ and indentation depth $d(t)$) was triggered at the start of the force modulation, where an oscillatory rotation of the external magnetic field and an power modulation of the exciting laser were applied for calibrations (see Supplementary Figure S2 for the sequence of the measurements). Typical AFM and confocal data of an indentation in PDMS are shown as functions of time in the left panel of Supplementary Figure S7. The data (measured in 30 s) are displayed after averaging of the continuous segments with 0.2 s duration to suppress the shot noise of the fluorescence data. By applying the Fourier transformation, the AFM local data and the ND fluorescence (represented by $x(t)$) are transformed to the frequency domain with angular frequency f' as,

$$\tilde{x}(f') \equiv \frac{1}{T}\int_0^T x(t)e^{-i2\pi f' t}\,\mathrm{d}t \tag{S1}$$

where $\tilde{x}$ is a complex function and T is the total measurement time. Examples of the data in frequency domain are shown in the right panel of Supplementary Figure S7. The colour dashed lines in the Supplementary Figure S7 indicate the first and high order modulation frequencies of force, magnetic field and laser power. Peaks are obtained in the AFM data at the force modulation frequency f (10 Hz, indicated by green dashed lines) and the corresponding high-order harmonics (2 to $4f$, also the green ones), while multiple peaks are given in the fluorescence data corresponding to the modulations of the force (f), magnetic field ($f_{\mathrm{B}} =$ 25 Hz, the purple ones) and laser ($f_{\mathrm{L}} = 65$ Hz, the cyan ones). Two corrections of the ODMR signals and the AFM signals were applied as follows.

Normalization of ODMR signals: By normalizing the on-resonance fluorescence with the off-

resonance one, the two-point ODMR signals can be obtained as $S \equiv (P_{\mathrm{on}} - P_{\mathrm{off}})/P_{\mathrm{off}}$. However, since the (linear) dependence of the fluorescence of NV centers on the laser power p_{L} is different with the on- and off-resonance MWs applied (denoted as $\partial_{p_{\mathrm{L}}} P\big|_{\mathrm{on/off}}$)[1], the laser power fluctuation (including the one induced by the laser reflected to ND from the AFM cantilever with modulation frequency f) cannot be eliminated by the simple normalization. Hence, a modified normalization is applied as $S \equiv (P_{\mathrm{on}} - \eta P_{\mathrm{off}})/(\eta P_{\mathrm{off}})$, where the factor η is the ratio between the two linear dependences as $\eta = \partial_{p_{\mathrm{L}}} P\big|_{\mathrm{on}} / \partial_{p_{\mathrm{L}}} P\big|_{\mathrm{off}}$. To obtain this factor η, a laser modulation is applied in the ODMR measurement with frequency f_{L} (see Supplementary Figures S1 and 2). The factor η is then obtained as $\eta = |\tilde{P}_{\mathrm{on}}(f_{\mathrm{L}})/\tilde{P}_{\mathrm{off}}(f_{\mathrm{L}})|$, which is the ratio of the amplitude of the laser modulation peaks for the on-resonance to the off-resonance fluorescence (indicated by cyan dashed lines in Supplementary Figures S7(c) and (d). Supplementary Figure S7(e) shows the normalized two-po int ODMR signal. The correction on laser fluctuation is valid by the elimination of the peak at f_{L} in the Fourier transform data ($\tilde{S}(f_{\mathrm{L}}) \approx 0$, see the right panel).

Hydrodynamic drag correction: The force modulation captured by the AFM includes the loading $L(t)$ on the soft samples (i.e. PDMS and cells) and the hydrodynamic drag on the oscillating cantilever in liquid environments[2]. To eliminate the effect of hydrodynamic drag and extract the loading on the soft samples, we calibrated the drag coefficient in the PDMS and live cell experiments as illustrated in Supplementary Figure S8. In the calibration, the force response $\tilde{F}_d(f)$ of the cantilever to small height oscillations $\tilde{h}_d(f)$ at different frequency f in the liquid environment approaching to (but not contacting) the sample with tip-sample separation d was measured as schemed in Supplementary Figure S8(a). Typical AFM data are plotted in Supplementary Figure S8(b) for the DNP-10-A tip (in the PDMS experiments). The drag coefficient is deduced by the slope $\mathrm{Im}[\tilde{F}_d(f)/\tilde{h}_d(f)]$ as a function of f for different d

[see Supplementary Figure S8(c)]. Supplementary Figure S8(d) plot the drag coefficient as a function of the tip-sample separation d. Such a function was fitted by the scaled spherical model of the cantilever,

$$b(d) = \frac{6\pi\epsilon a_{\text{eff}}^2}{d + d_{\text{eff}}}, \tag{S2}$$

where ϵ is the dynamic viscosity of the liquid and a_{eff} and d_{eff} are the two fitting parameters accounting for the effective cantilever geometry. The red line in Supplementary Figure S8(c) is the fitting results. Similar calibration are shown in Supplementary Figures S8(e) to (g) for the PFQNM-LC-A-CAL tip (in the cell experiments). The drag coefficients b_0 for the DNP-10-A tip (in the PDMS experiments) and PFQNM-LC-A-CAL tip (in the cell experiments) were calibrated to be 1.0×10^{-5} and 8.7×10^{-7} N s m^{-1} in the limit $d \to 0$. Hence, the loading of the indentation was obtained by the AFM data after correction as,

$$\tilde{L}(f) = \tilde{F}(f) - i2\pi f b_0 \tilde{d}(f). \tag{S3}$$

Supplementary Figure S7(f) shows a typical resultant loading $\tilde{L}(f)$ of the indentation.

The corrected loading $L(t)$, the indentation depth $d(t)$ and the normalized ODMR signal $S(t)$ are plotted in Fig. 2(c) in the main text after averaging. The amplitude and the phase of different modulations $x(t)$ were extracted as the absolute value and the argument of the complex number $\tilde{x}(f)$ (see Supplementary Figures S13 and S18 for typical loading and depth amplitudes in the PDMS and live cell experiments, respectively). The amplitude of the oscillatory ND rotation was deduced by

$$|\tilde{\chi}| = \left|\frac{\tilde{S}(f)}{\tilde{S}(f_{\text{B}})}\right| |\tilde{\chi}_{\text{B}}|, \tag{S4}$$

where $|\tilde{\chi}_{\text{B}}|$ is the known amplitude of the magnetic field modulation controlled by the current

of the coil (see Supplementary Figure S4). Finally, the local and nonlocal phases are evaluated as,

$$\phi_{\mathrm{AFM}} = \arg\left[\frac{\tilde{L}(f)}{\tilde{d}(f)}\right] \quad \text{and} \quad \phi_{\mathrm{ND}} = \arg\left[\frac{\tilde{L}(f)}{\tilde{S}(f)}\right], \tag{S5}$$

Reference

(1) Dréau, A.; Lesik, M.; Rondin, L.; Spinicelli, P.; Arcizet, O.; Roch, J.-F.; Jacques, V. Avoiding Power Broadening in Optically Detected Magnetic Resonance of Single NV Defects for Enhanced Dc Magnetic Field Sensitivity. *Physical Review B* **2011**, *84* (19), 195204. https://doi.org/10.1103/PhysRevB.84.195204.
(2) Rother, J.; Nöding, H.; Mey, I.; Janshoff, A. Atomic Force Microscopy-Based Microrheology Reveals Significant Differences in the Viscoelastic Response between Malign and Benign Cell Lines. *Open Biology 4* (5), 140046. https://doi.org/10.1098/rsob.140046.
